# Knockdown of SLC39A14 inhibits glioma progression by promoting erastin-induced ferroptosis *SLC39A14 knockdown inhibits glioma progression*

**DOI:** 10.1186/s12885-023-11637-0

**Published:** 2023-11-17

**Authors:** Yunwen Zhang, Xinghai Wu, Jiyong Zhu, Ruibin Lu, Yian Ouyang

**Affiliations:** 1 Department of Neurosurgery, First Clinical Medical College of Gannan Medical University, No.1 Xueyuan Road, Zhanggong District, Ganzhou City, 341000 Jiangxi Province China; 2 Department of Neurosurgery, Zhangye People’s Hospital Affiliated to Hexi University, No. 67 Xihuan Road, Ganzhou District, Zhangye City, 734000 Gansu Province China; 3 Department of Neurosurgery, Guilin Municipal Hospital of Traditional Chinese Medicine, Guangxi Zhuang Autonomous Region, No. 2 Lingui Road, Xiangshan District, Guilin City, 541002 China; 4 Department of Neurosurgery, First Affiliated Hospital of Gannan Medical University, No.23 Qingnian Road, Zhanggong District, Ganzhou City, 341000 Jiangxi Province China

**Keywords:** Glioma, SLC39A14, Ferroptosis, The cGMP-PKG signaling pathway

## Abstract

**Background:**

Ferroptosis is a newly classified form of regulated cell death with implications in various tumor progression pathways. However, the roles and mechanisms of ferroptosis-related genes in glioma remain unclear.

**Methods:**

Bioinformatics analysis was employed to identify differentially expressed ferroptosis-related genes in glioma. The expression levels of hub genes were assessed using real-time reverse transcriptase-polymerase chain reaction (RT-qPCR). To explore the role of SLC39A14 in glioma, a series of in vitro assays were conducted, including cell counting kit-8 (CCK-8), 5-ethynyl-2’-deoxyuridine (EdU), flow cytometry, wound healing, and Transwell assays. Enzyme-linked immunosorbent assay (ELISA) was utilized to measure the levels of indicators associated with ferroptosis. Hematoxylin-eosin (HE) and immunohistochemistry (IHC) staining were performed to illustrate the clinicopathological features of the mouse transplantation tumor model. Additionally, Western blot analysis was used to assess the expression of the cGMP-PKG pathway-related proteins.

**Results:**

Seven ferroptosis-related hub genes, namely SLC39A14, WWTR1, STEAP3, NOTCH2, IREB2, HIF1A, and FANCD2, were identified, all of which were highly expressed in glioma. Knockdown of SLC39A14 inhibited glioma cell proliferation, migration, and invasion, while promoting apoptosis. Moreover, SLC39A14 knockdown also facilitated erastin-induced ferroptosis, leading to the suppression of mouse transplantation tumor growth. Mechanistically, SLC39A14 knockdown inhibited the cGMP-PKG signaling pathway activation.

**Conclusion:**

Silencing SLC39A14 inhibits ferroptosis and tumor progression, potentially involving the regulation of the cGMP-PKG signaling pathway.

**Supplementary Information:**

The online version contains supplementary material available at 10.1186/s12885-023-11637-0.

## Introduction

Glioma is the predominant type of primary carcinoma that develops within the central nervous system [1]. It is associated with a high mortality rate and poor prognosis, particularly in high-grade cases, with an average survival of no more than 14.6 months [2]. Currently, the main treatment options for glioma include chemotherapy, surgery, radiotherapy, and immunotherapy [3–5]. However, the diffuse and aggressive nature of glioma, along with the brain tumor barrier, pose challenges of drug and systemic therapy [6]. Consequently, it is necessary to uncover the mechanisms of glioma progression and pinpoint novel therapeutic targets.

Ferroptosis is an emerging type of programmed cell death distinguished by disrupted iron metabolism, lipid metabolism, increased oxidative stress, and depletion of glutathione (GSH) [7]. It plays a significant role in the advancement of various diseases, including neurodegenerative diseases [8], cardiovascular disease [9], and cancer [10]. Researches have shown that several tumor-associated factors, such as p53 and BRCA1, influence tumor progression by participating in ferroptosis [11, 12]. Furthermore, ferroptosis holds promise as a potential strategy to counteract drug resistance mechanisms in conventional cancer treatments by regulating radiotherapy, chemotherapy, and immunotherapy [13]. In glioma, studies have shown that inhibiting ferroptosis promotes malignant transformation, increases proliferation, and enhances angiogenesis [14, 15]. Mechanistically, multiple genes associated with ferroptosis have been recognized to be implicated in glioma development. For example, SLC1A5 amplifies malignant traits by influencing ferroptosis and the immune microenvironment in glioma [16]. Models constructed from ferroptosis-related genes can be used for prediction of clinical outcomes and indication of treatment in gliomas [17]. However, the mechanisms of ferroptosis in glioma are complex, and further research is needed for a comprehensive understanding.SLC39A14, a member of the SLC39A family, has transport characteristics [18]. It mediates the transport of metals other than zinc, including manganese and iron, due to its substitution of the first histidine residue in the conserved motif of HEXPHE for glutamic acid [19]. SLC39A14 is an essential transporter of nontransferrin-bound iron and mediates cellular uptake of non-transferrin-bound iron [20]. Moreover, SLC39A14 plays an important role in tumor progression by regulating ferroptosis. Study has revealed that SLC39A14 is a key regulator of ferroptosis in a variety of tumors, such as head and neck squamous cell carcinoma and esophageal squamous cell carcinoma [21, 22]. However, the role of SLC39A14 and the mechanisms regulating ferroptosis in gliomas have been scarcely reported.

In this research, our objective was to identify the pivotal genes associated with ferroptosis in glioma. We initially analyzed two datasets, namely GSE15209 and GSE31262, to pinpoint the differentially expressed genes (DEGs) in glioma. Following that, we performed an intersection analysis to identify the DEGs that were linked to ferroptosis in glioma. SLC39A14 was identified as a key factor through bioinformatics analysis. Subsequently, we delved deeper into investigating the function of SLC39A14 in glioma and sought to elucidate the precise molecular mechanisms that connected with ferroptosis. In vitro experiments revealed that the knockdown of SLC39A14 significantly inhibited glioma development and promoted erastin-induced ferroptosis. In vivo experiments further confirmed that the knockdown of SLC39A14 inhibited tumor growth by promoting ferroptosis. Mechanistically, it was found that the knockdown of SLC39A14 led to the regulation of the cGMP-PKG signaling pathway.

## Materials and methods

### Acquisition of microarray data and identification of DEGs

A search for the keyword “Glioma” led to the identification of two datasets, GSE15209, and GSE31262, in the Gene Expression Omnibus (GEO) database(https://www.ncbi.nlm.nih.gov/geo/), which were used for the following research. The dataset GSE15209 ([HG-U133_Plus_2] Affymetrix Human Genome U133 Plus 2.0 Array) contains six normal samples and six glioma samples. The GSE31262 dataset (ABI Human Genome Survey Microarray Version 2) includes five normal samples and nine glioma samples. The GEO2R tool (https://www.ncbi.nlm.nih.gov/geo/geo2r) was used to perform differential expression analysis on the gene expression data.The screening criterion for DEGs was *p* ≤ 0.05 and |log FC| ≥ 0.1. The ggplot2 and pheatmap packages in R software (version 3.6.0) were utilized to generate volcano plots and heatmaps of the DEGs.

### Identification of ferroptosis-related DEGs and functional enrichment analysis

Genes involved in ferroptosis were screened from the Coxpresdb database (https://coxpresdb.jp/). Overlapping DEGs between the GSE15209 and GSE31262 datasets and the genes associated with ferroptosis were investigated using the Venn diagram tool (http://bioinformatics.psb.ugent.be/webtools/Venn/). The DEGs related to ferroptosis were analyzed for Gene Ontology (GO) and Kyoto Encyclopedia of Genes and Genomes (KEGG) enrichment using the DAVID database (Database for Annotation, Visualization, and Integrated Discovery; https://david.ncifcrf.gov/). The GO analysis involves molecular function (MF), cellular component (CC), and biological process (BP).

### Protein-protein interaction (PPI) network construction and key genes identification

A PPI network was constructed by applying the STRING database (https://string-db.org/). Subsequently, in Cytoscape software (version 3.6.1), The PPI network was visualized. Then filtering of key modules was performed utilizing the Molecular Complex Detection (MCODE) plugin in Cytoscape software. The selection criteria included a degree cutoff of 2, a node score cutoff of 0.2, a k-score of 2, and a maximum depth of 100. Afterward, the key genes were identified using the cytoHubba plugin, which offers various algorithms and methods to assess the importance and centrality of genes within the key module.

### Bioinformatics analysis of key genes

The analysis of the expression, principal component analysis (PCA), GO, and receiver operating characteristic (ROC) curve analysis of seven key genes was conducted based on the GSE15209 dataset. The ggridges package in the R software was used to visualize the gene expression ridgelines. PCA was performed utilizing the factoextra package in R software. The chord diagram representing the GO enrichment of the hub genes was generated with the circlize package in R software. For evaluating the precision of the hub genes in diagnosing glioma, ROC curve analysis was conducted with the pROC package. To further analyze the expression of key genes in tumor tissues, we utilized the GEPIA 2 database (http://gepia2.cancer-pku.cn/#index). Additionally, the GEPIA 2 database was employed to examine the association between SLC39A14 expression and overall survival of patients with glioma. The TIMER database (https://cistrome.shinyapps.io/timer/) was applied to investigate the relationship between SLC39A14 expression and the infiltration of immune cells.

### Cell culture

Normal brain tissue cells (HEB) and glioma cells (U251 and LN229) were acquired from Icellbioscience Biotechnology Co., Ltd (Shanghai, China). The cells were cultivated in DMEM medium containing 10% fetal bovine serum (Thermo Fisher Scientific, Massachusetts, USA) and 10 mg/mL penicillin and streptomycin, and placed in an incubator at 37℃ with 5% CO_2_.

### Cell transfection

All the plasmids and RNA fragments were acquired from Genepharma Biotechnology Co., Ltd (Shanghai, China). A total of 1 × 10^5^ cells were inoculated into each well in six-well plates and allowed to grow until reaching 30–50% confluence. Transfection was performed by introducing siRNA/NC (50 nM) into the cells using Lipofectamine 3000 (Thermo Fisher Scientific). The transfection efficiency was assessed through the reverse transcription-quantitative polymerase chain reaction (RT-qPCR) analysis after 48-hour. The RNA sequences are displayed in Supplementary Table 1.

### RT-qPCR

To isolate total RNA, Trizol reagent (Thermo Fisher Scientifi) was used. The extracted RNA was converted to complementary DNA adhering to the instructions provided by the reverse transcription kit (Thermo Fisher Scientific). Then RT-qPCR analysis was performed on the obtained complementary DNA using the ABI 7500 RT-PCR system (Thermo Fisher Scientific) with the SYBR Premix Ex Taq (Takara, Dalian, China). In accordance with the 2^−ΔΔCT^ method, the gene expression levels were normalized to the Glyceraldehyde 3-phosphate dehydrogenase (GAPDH). Supplementary Table 1 displays the detailed sequences.

### Cell counting Kit-8 assay (CCK-8)

In each well, a total of 1 × 10^5^ cells were seeded and incubated for 0, 24,48, 72, and 96 h, respectively. At the designated endpoint, 10 µl of CCK8 solution was introduced into each well. The optical density (OD) value was obtained at a wavelength of 450 nm using a microplate reader (DALB, Shanghai, China).

### 5-ethynyl-2’-deoxyuridine (EdU) assay

The cells were planted in 24-well plates at a concentration of 1 × 10^5^ cells per well and cultivated for 24 h. To analyze and evaluate cell proliferation, the EdU analysis kit (Beyotime Biotechnology, Shanghai, China) was employed. The specific method for EdU analysis was conducted following the guidelines given by the manufacturer. Under a microscope (Olympus, Tokyo, Japan), images were captured. The percentage of EDU-positive cells was computed using the formula: EDU‐positive cell count/total cell count × 100%.

### Transwell assay

Cells (1 × 10^5^) were plated in the upper chamber with 100 µL of serum-free medium. In the lower chamber, 600 µL of medium supplemented with 20% FBS was used as a chemoattractant. After incubating for 48 h, the cells in the upper chamber were removed by gently wiping with a cotton swab. Cells that had invaded and attached to the underside of the membrane were then treated and stained with 0.1% crystal violet. Subsequently, the invaded cells were counted in five randomly chosen microscopic areas under 200× magnification.

### Wound healing assay

When the cells reached 80–90% confluence in six-well plates, we gently made an incision in the monolayer using a pipette tip to generate a mechanical scratch. Imaging of the wound was performed using a microscope (Olympus) at both 0 and 24 h. The ImageJ software was utilized to measure the widths of the wounds.

### Flow cytometry

To evaluate apoptosis, apoptotic cells were treated with 50 µg/mL Annexin V-FITC (Abcam, Cambridge, UK) and 10 µg/mL propidium iodide (PI; Thermo Fisher Scientific) for 10 min at a temperature of 37 °C. Subsequently, the stained cells were subjected to apoptosis analysis using a FACScan flow cytometer (Becton, Dickinson and Company, New Jersey, USA).

### Preparation of erastin and ferrostatin-1 (fer-1) solutions

Erastin is the first identified ferroptosis activator [23]. Additionally, fer-1 is an effective ferroptosis inhibitor, demonstrating higher efficiency than phenolic antioxidants [24, 25]. In this study, U251 and LN229 cells were treated with erastin and fer-1 to activate or inhibit ferroptosis. Erastin and fer-1, obtained from Yuanye Biological Co., Ltd (Shanghai, China), were dissolved in dimethyl sulfoxide (DMSO) to create stock solutions and stored at -20 °C. U251 and LN229 cells with silenced SLC39A14 were treated with erastin (10 µM) and fer-1 solutions (2 µM) either in combination or individually for 48 h.

### Enzyme-linked immunosorbent assay (ELISA)

ELISA kits (Abcam, Cambridge, UK) was conducted to assess the levels of GSH, Malondialdehyde (MDA), Fe^2+^, and cGMP. Measurements of the absorbance for each sample were taken using a microplate reader (DALB).

### Western blot

Complete protein extraction was performed utilizing pre-cooled RIPA buffer (Solarbio, Beijing, China) supplied with protease inhibitors (Thermo Fisher Scientific). Cellular proteins, with a quantity of 20 µg, were separated on a sodium dodecyl sulfate-polyacrylamide gel electrophoresis. Then, proteins were transferred to polyvinylidene fluoride (PVDF) membranes (Roche, Basel, Switzerland). PVDF membranes were treated with 5% skim milk to inhibit nonspecific binding. Following that, the membranes were incubated with primary antibodies, including SLC39A14 (1:2000, Abcam), GPX4 (1:2000, Abcam), NRF2 (1:2000, Abcam), SLC7A11 (1:2000, Abcam), sGC (1:2000, Abcam), PKG1 (1:2000, Abcam), PKG2 (1:2000, Abcam), and GAPDH (1:2000, Abcam). The primary antibodies were then incubated with the corresponding secondary antibodies. The immunoblots were visualized using an ECL luminescent solution (Amersham, Little Chalfont, UK).

### Construction of xenograft Tumor mouse models

All female BALB/c nude mice (6–8 weeks old, 18–20 g) were acquired from SPF (Beijing) Biotechnology Co., Ltd. A total of 18 mice were randomly allocated into three groups (*n* = 6 per group): Saline + LV-NC, SAS + LV-NC, and SAS + LV-shSLC39A14 groups. For SAS treatment, 8 mg SAS was dissolved in 0.2 ml Saline. mice were administered intraperitoneally twice daily for 7 days. U251 cells were reconstituted in PBS at a concentration of 2 × 10^5^ cells/100 µL and then subcutaneously administered into the armpits of mice. Tumor size was assessed every seven days by using calipers to measure the length and width, and tumor volumes were computed using the formula: V = 0.5 × L (length) × W^2^ (width). On the 28th day after transplantation, all nude mice were euthanized by the intraperitoneal administration of 120 mg/kg ketamine and 150 mg/kg xylazine (2:1 solution), and the tumors were weighed. All animal experiments conducted in this study were approved by the Animal Experimentation Ethics Committee.

### Immunohistochemistry (IHC)

The tissue section was subjected to the following steps: baking, deparaffinization, hydration, and antigen retrieval in citrate buffer with a pH of 6.0. The sections underwent conventional dewaxing to remove paraffin. Subsequently, endogenous peroxidase activity was blocked by treating with 3% hydrogen peroxide for 10 min. Subsequent to rinsing with PBS, the slices were incubated with a blocking solution for 15 min. The sections were exposed to Ki67 primary antibody (1:100; Abcam).and incubated at room temperature for 1 h. Following three washes with PBS, the sectionswas subjected to a 1-hour incubation with secondary antibodies at room temperature. DAB (3,3’-diaminobenzidine) was employed as the chromogen. Afterward, the slide underwent hematoxylin counterstaining and was sealed following routine dehydration. Subsequently, photographs of the slide were examined using an optical microscope (Olympus).

### Hematoxylin-Eosin (HE) staining

The heterotransplanted tumors were preserved by immersing them in a 4% paraformaldehyde solution and then embedded in paraffin. Subsequently, 4 μm sections were sliced from the paraffin blocks and stained using HE staining. The stained sections were analyzed under a light microscope at 200× magnification.

### Statistical analysis

All data were gathered from at least three independent measurements and were expressed as mean ± standard deviation (SD). The statistical analysis included the use of the student’s t-test for comparing two groups. For comparisons among multiple groups, one-way ANOVA with Tukey’s multiple comparisons tests was applied. Statistical significance was considered when the p-value was less than 0.05.

## Result

### Identification of DEG in gliomas

**Fig. 1:**
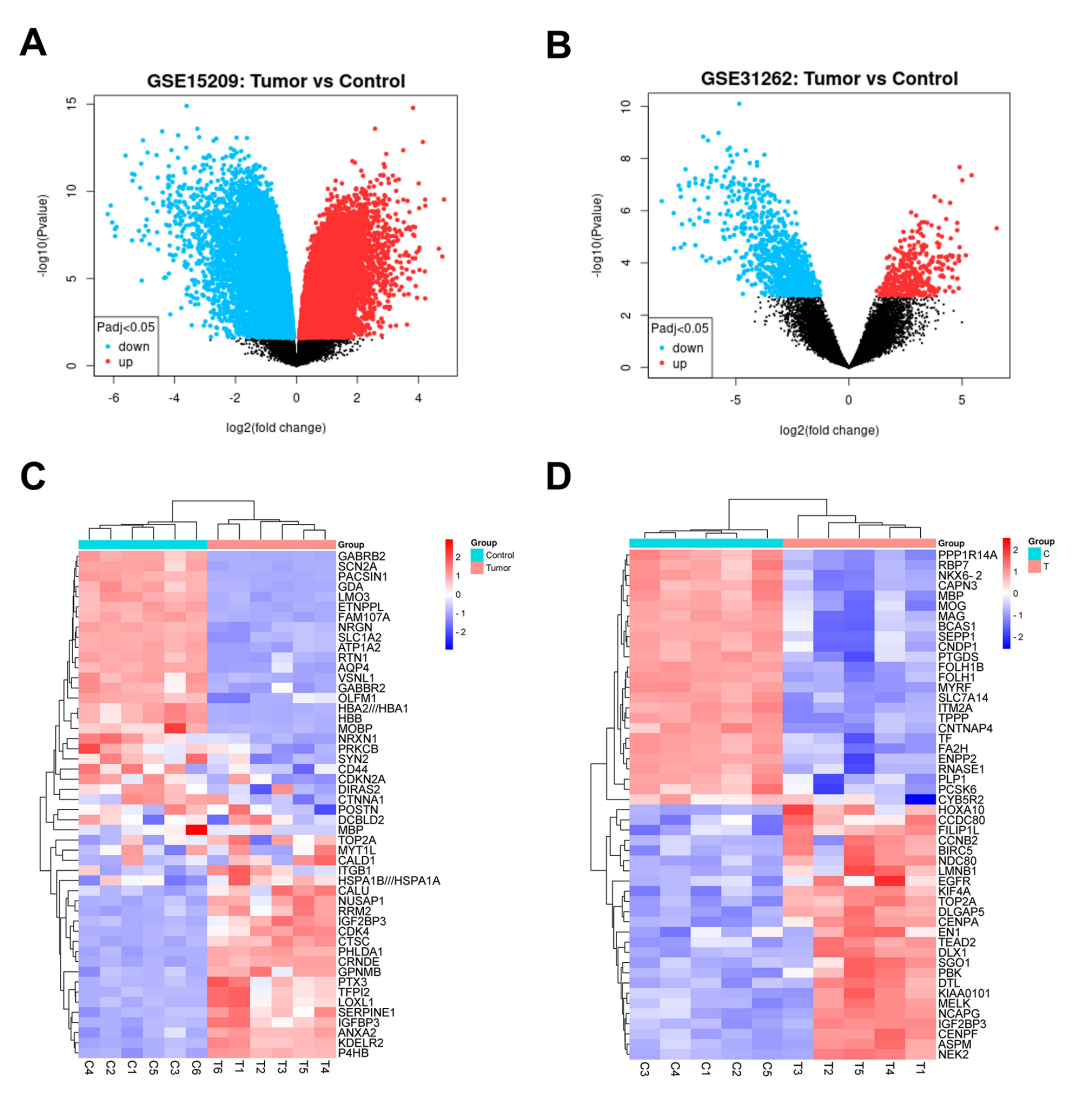
Identification of DEG in gliomas. **A-B**. Volcano plots of DEGs in the GSE15209 and GSE31262datasets (*p* ≤ 05 and |log 2 FC|  ≥  1.0). The red nodes indicate upregulated genes, while the blue nodes indicate downregulated genes. **C-D**. Heatmaps of DEGs in two datasets. Given the extensive number of DEGs, the heatmap displays the top 15 upregulated and downregulated genes

The sample data from GSE15209 and GSE31262 were analyzed using the GEO2R tool, applying a selection criterion of p-value ≤ 0.05 and |log 2 FC|  ≥  1.0. In the GSE15209 dataset, a total of 4655 DEGs were identified, while the GSE31262 dataset revealed 2940 DEGs. The volcano plot (Fig. 1A and B) visually represented the DEGs from both datasets. Additionally, the heatmaps (Fig. 1 C and 1D) displayed the top 15 significant DEGs obtained from the GSE15209 and GSE31262 datasets (Supplementary Tables 2 and 3), revealing notable differences in the DEGs between the control group and the tumor group.

### Identification ferroptosis-related common DEGs and functional enrichment analysis

**Fig. 2:**
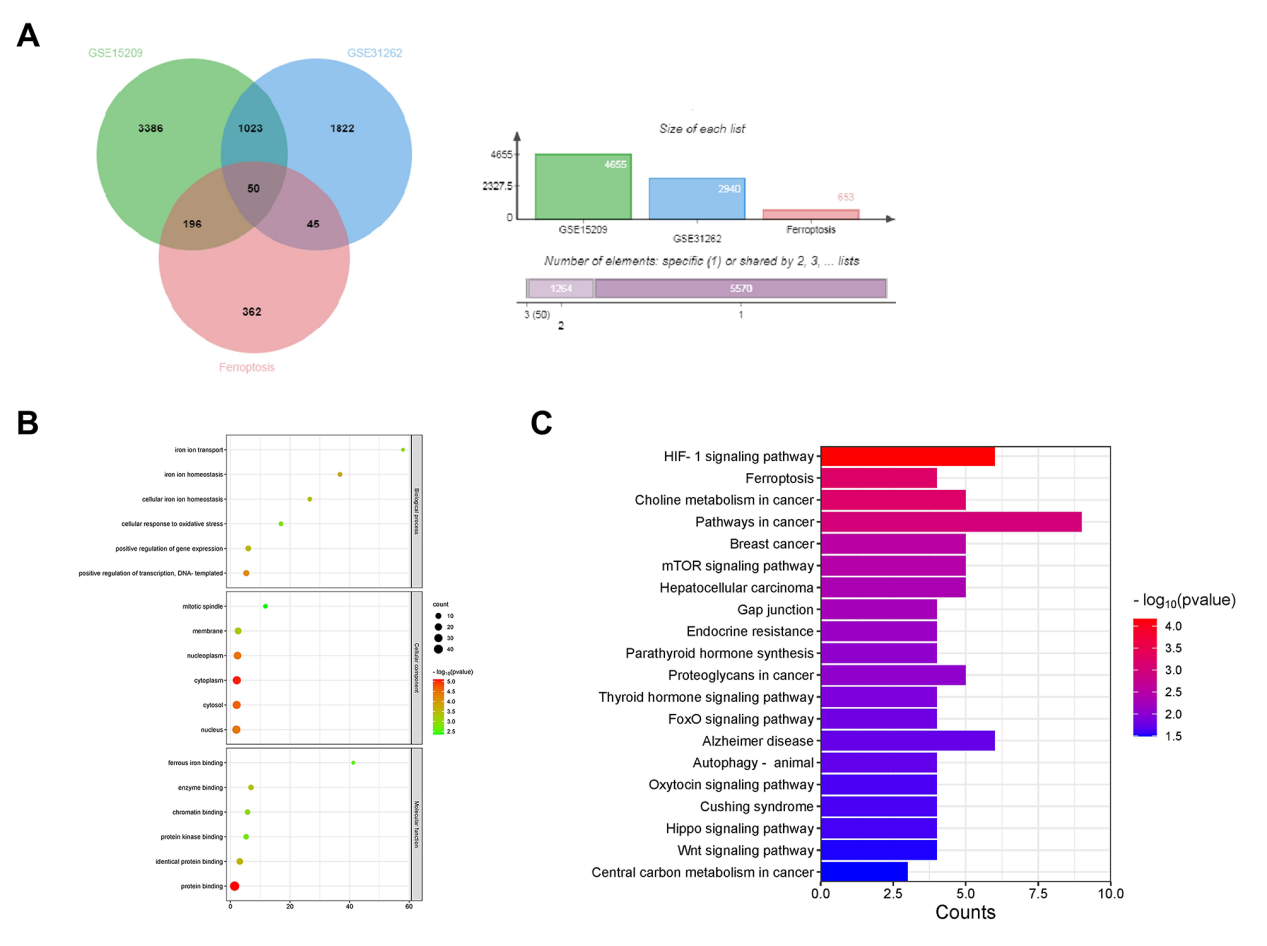
Identification ferroptosis-related overlapping DEGs and functional enrichment analysis. **(A)** Venn diagram illustrated the intersection of the overlapping 50 ferroptosis-related DEGs in the three datasets. **(B)** The top six GO terms of DEGs, including BP, CC, and MF, were shown in the bubble chart. **(C)** Histogram of KEGG pathways for the top 20 of DEGs, mainly included HIF-1 signaling pathway, ferroptosis, choline metabolism in cancer, pathways in cancer, and breast cancer, etc [74–76]

The DEGs in the GSE15209 and GSE31262 datasets were then intersected with ferroptosis-related genes taken from the Coxpresdb database to screen DEGs associated with ferroptosis in glioma. The Venn diagram showed that there was a total of 50 ferroptosis-related overlapping DEGs in gliomas (Fig. 2A). To explore the potential biological functions of these DEGs, we performed GO and KEGG functional enrichment. The top six most important GO terms were shown in Supplementary Table 4. In the BP category, overlapping DEGs were mainly participated in positive regulation of transcription, DNA-templated, positive regulation of gene expression, and cellular response to oxidative stress. In the CC category, DEGs were primarily linked to nucleus, cytoplasm, and cytosol. In MF category, the DEGs were mainly involved in protein binding, identical protein binding, and protein kinase binding (Fig. 2B). The analysis of KEGG pathway (Supplementary Table 5) indicated that the DEGs were primarily participated in the HIF-1 signaling pathway, ferroptosis, choline metabolism in cancer, pathways in cancer, and breast cancer, suggesting that DEGs screened out play an important role in tumor development (Fig. 2C).

### Identification of the hub gene SLC39A14

**Fig. 3:**
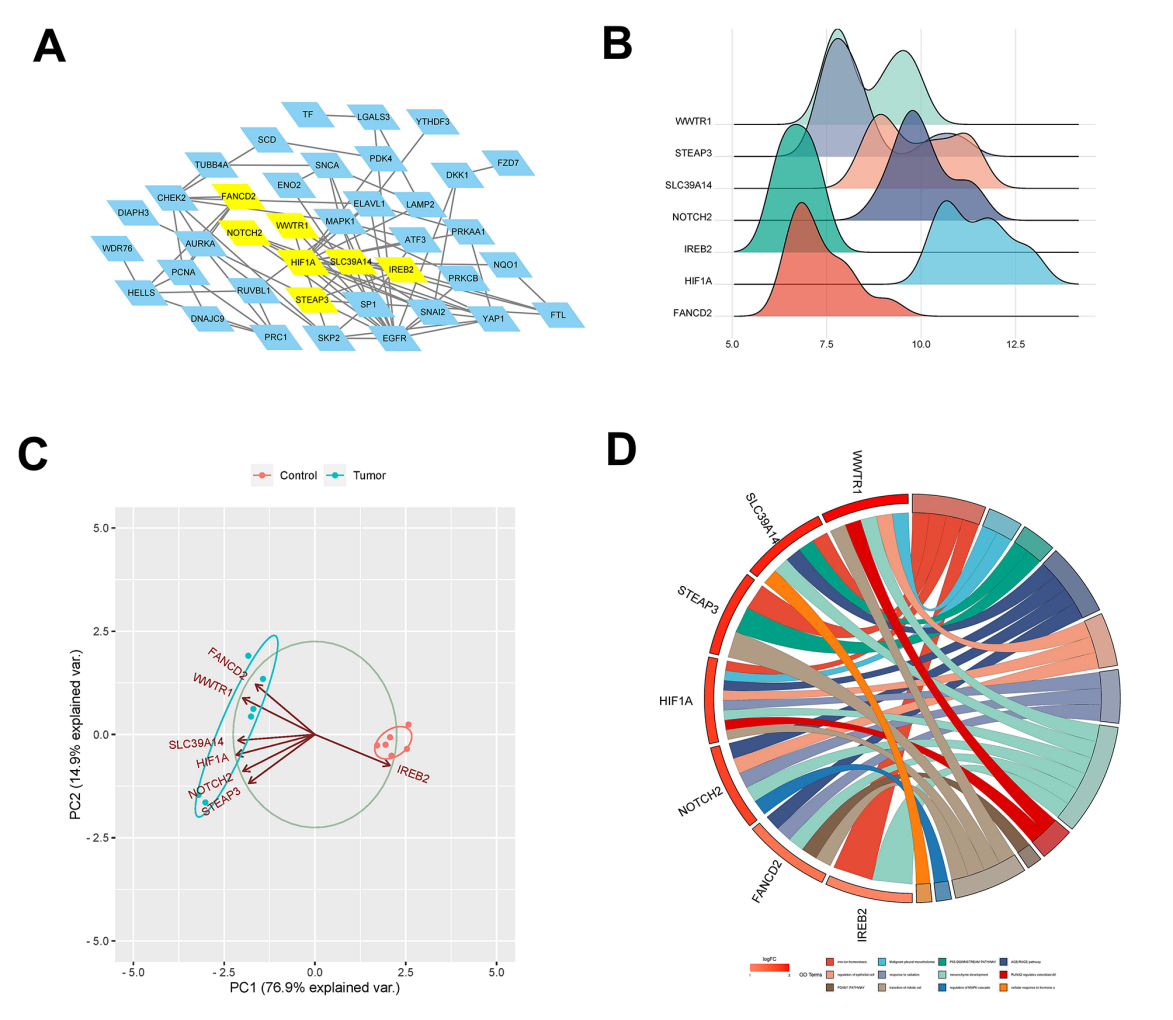
Screening and analysis of key genes. **(A)** Key genes screened from key modules in Cytoscape software via CytoHubba plugin. **(B)** A ridgeline map of the expression patterns of seven key genes. The horizontal coordinate represents the amount of gene expression, the shape of the peak indicates the dispersion between a set of data, and its height represents the number of samples corresponding to the amount of gene expression. **(C)** PCA analysis of key genes and generated PC1 and PC2 axes. **(D)** GO-enriched chordal maps of key genes. On the left side of the graph, the hub genes are arranged in descending order based on their LogFC (logarithm of fold change). On the right side, the list of GO terms is presented according to their enrichment level. The color shades of the lines connecting the hub genes and GO terms indicate the number of variations

The STRING database was utilized to construct PPI networks of DEGs. Subsequently, the PPI network model was displayed using Cytoscape software. MCODE was used to identify key modules from the PPI network (Fig. 3A). Additionally, the CytoHubba plugin was employed to perform additional screening for hub genes within the significant module, leading to the identification of seven hub genes: WWTR1, STEAP3, SLC39A14, NOTCH2, IREB2, HIF1A, and FANCD2. Based on the samples in the GSE15209 dataset, the expression and sample distribution of the seven key genes were analyzed. The findings indicated that the HIF1A expression level was the highest, and WWTR1 exhibited the widest sample distribution (Fig. 3B). PCA analysis revealed that PC1 accounted for a significant portion of the data variance (76.9%), suggesting that PC1 captured the most significant patterns and differences among the samples. Although PC2 contributed less to the variance (14.9%), this principal component still provided valuable insights into the underlying data structure (Fig. 3C). GO-enriched chordal plots of the seven key genes were generated to illustrate the changes in the functional strength of the pathway (Fig. 3D).

**Fig. 4:**
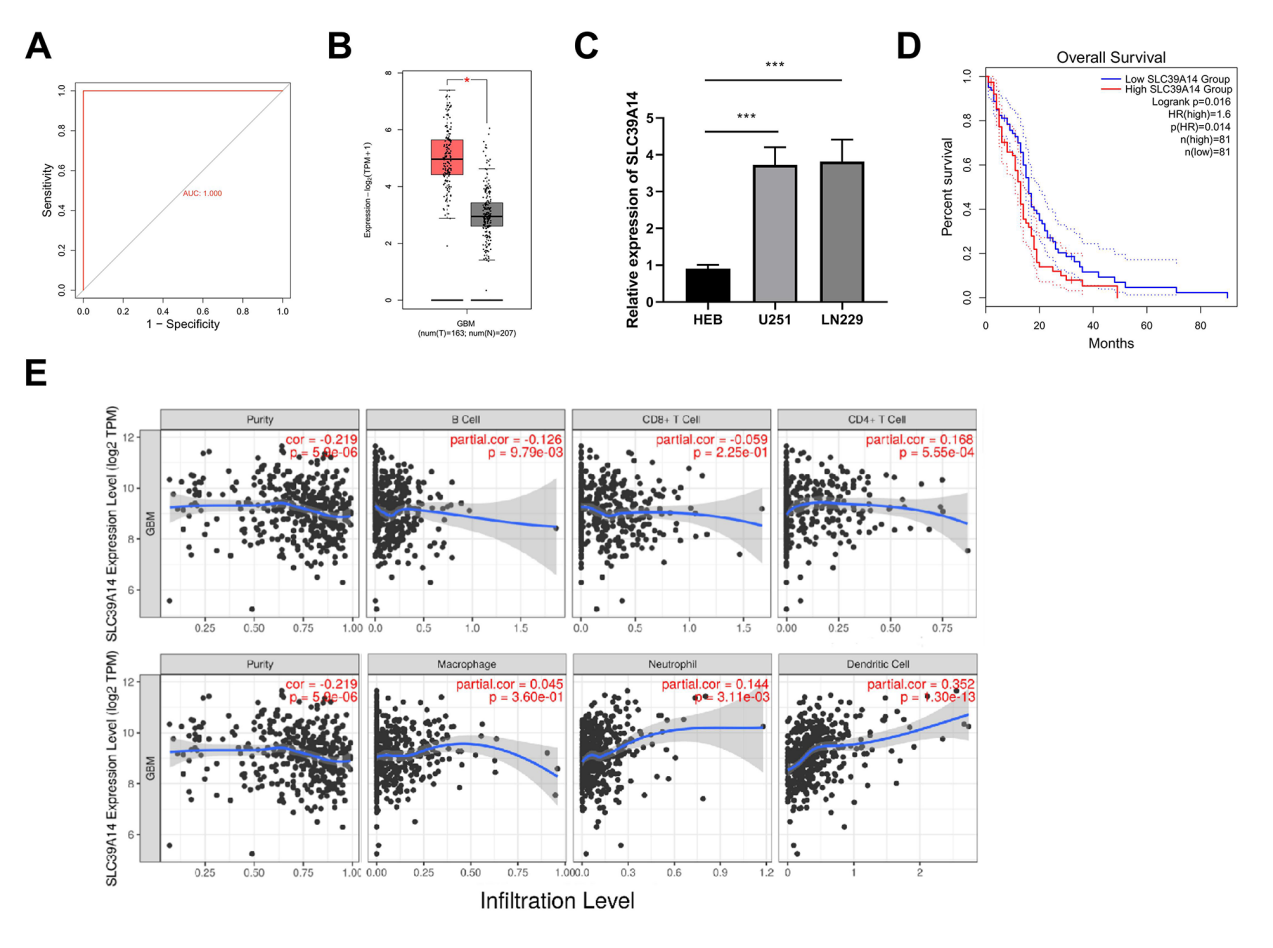
ROC curve, expression, survival and immune infiltration analysis of SLC39A14. **(A)** The AUC value of SLC39A14 was 1.000 and can be used as a diagnostic biomarker for glioma. **(B)** In the GEPIA 2 database, the mRNA expression level of SLC39A14 was significantly higher in tumors than in normal tissues. **(C)** The expression level of SLC39A14 was elevated in U251 and LN229 cells in comparison to HEB cells by RT-qPCR. **(D)** High expression of SLC39A14 predicted a poor prognosis for glioma patients. **(E)** The correlation between SLC39A14 and immune infiltrating cells was explored in the TIMER database

Subsequently, ROC curve analysis suggested that the AUC values of WWTR1, STEAP3, SLC39A14, NOTCH2, IREB2, HIF1A, and FANCD2 were all 1.000, indicating their potential as diagnostic biomarkers for gliomas (Fig. 4A and Supplementary Fig. 1). In the GEPIA 2 database, substantially upregulation of the seven key genes in tumor tissues compared to normal tissues was observed, suggesting they likely function as oncogenes in gliomas (Fig. 4B and Supplementary Fig. 2). The expression level of seven hub genes in glioma cells was further examined, revealing elevated expression in U251 and LN229 cells compared to HEB cells (Fig. 4C and Supplementary Fig. 3). Survival analysis revealed that the overexpression of SLC39A14 significantly suppressed the possibility of patient survival (Fig. 4D). So SLC39A14 was selected for the follow-up study. Subsequently, the relationship between SLC39A14 and immune infiltration was evaluated in the TIMER database, which demonstrated a strong positive correlation between SLC39A14 expression and dendritic cells (Fig. 4E).

### Knockdown of SLC39A14 suppressed glioma cell proliferation, invasion and migration and promotes apoptosis

**Fig. 5:**
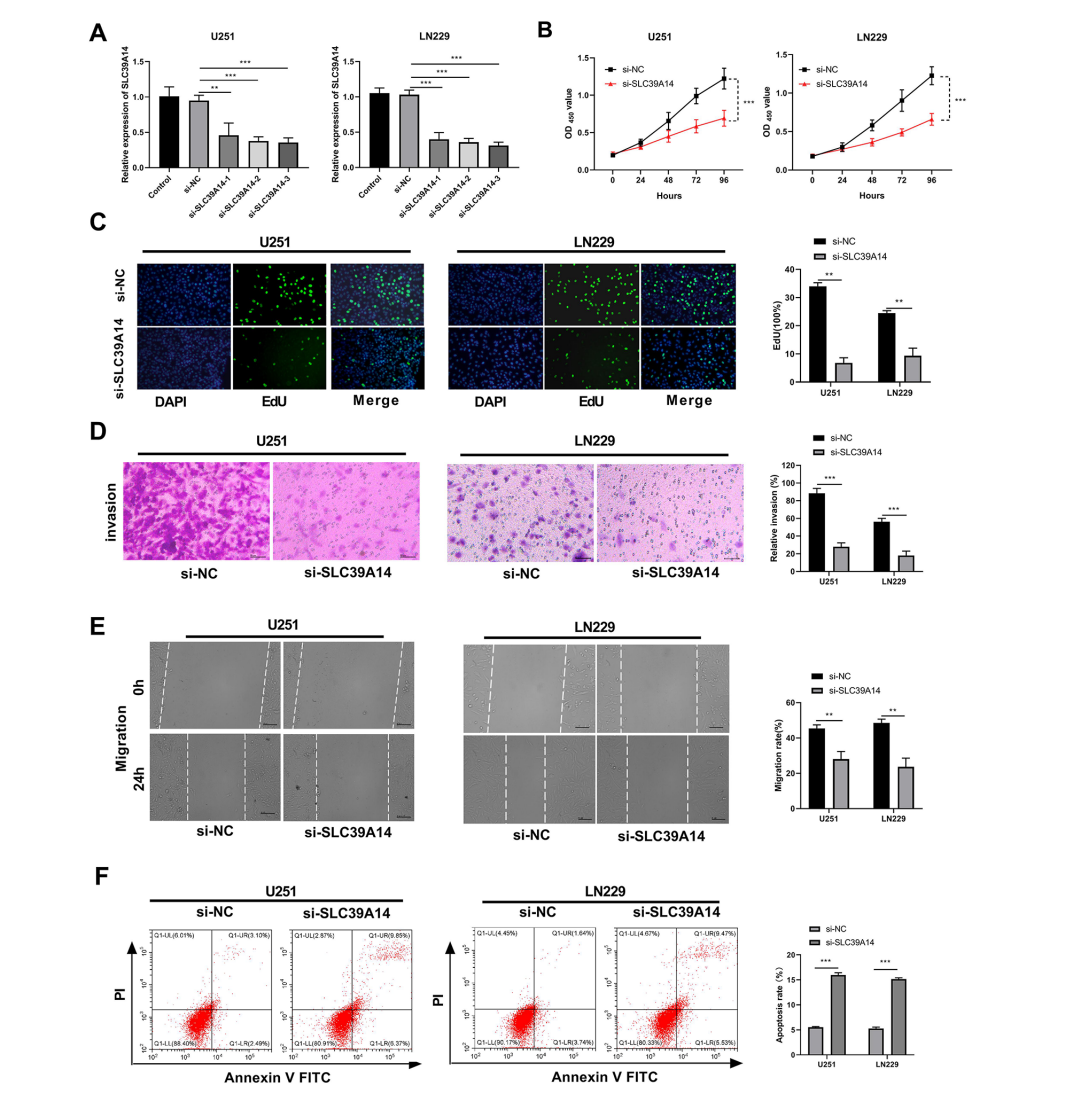
Silencing of SLC39A14 inhibited glioma cell proliferation, invasion and migration, while promoting apoptosis. **(A)** RT-qPCR was used to detect the transfection efficiency of si-SLC39A14 -1, si- SLC39A14 -2, and si- SLC39A14 -3. **(B)** The CCK-8 assay was used to measure the activity of U251 and LN229 cells in the si-NC and si- SLC39A14 groups. **(C)** The proliferation of U251 and LN229 cells in the si-NC and si-SLC39A14 groups was evaluated using the EdU assay. **(D)** To assess the effect of SLC39A14 knockdown on glioma cell invasion, the Transwell assay was conducted. **(E)** The migration of U251 and LN229 cells in the si-NC and si-SLC39A14 groups was measured using the Wound healing assay. **(F)** The impact of SLC39A14 knockdown on the apoptosis of glioma cells was assessed through flow cytometry. **P < 0.01; ***P < 0.001 vs. si-NC group

U251 and LN229 cells were assigned to five groups: Control, si-NC, si-SLC39A14-1, si-SLC39A14-2, and si-SLC39A14-3. Transfection efficiency of each group was assessed through RT-qPCR, which revealed that the expression level of SLC39A14 in the SLC39A14-silenced groups was significantly lower than that in the si-NC group. Among the SLC39A14-silenced groups, si-SLC39A14-3 demonstrated the highest silencing efficiency, thus it was selected for subsequent experiments (Fig. 5A). CCK-8 results demonstrated that cell viability of U251 and LN229 cells was significantly reduced in the si-SLC39A14 group compared to the si-NC group (Fig. 5B). Additionally, EdU results showed that silencing SLC39A14 remarkably impeded the proliferation of glioma cells (Fig. 5C). Furthermore, Transwell assay indicated a substantial reduction in the invasive ability of U251 and LN229 cells in the si-SLC39A14 group relative to the si-NC group (Fig. 5D). Wound healing also confirmed that reduced cell migration was a result of SLC39A14 knockdown (Fig. 5E). Finally, we evaluated the effect of SLC39A14 on apoptosis by flow cytometry, and the results indicated a considerable increase in apoptosis levels in the si-SLC39A14 group compared with the si-NC group (Fig. 5F).

### Knockdown of SLC39A14 promoted erastin-induced ferroptosis

**Fig. 6:**
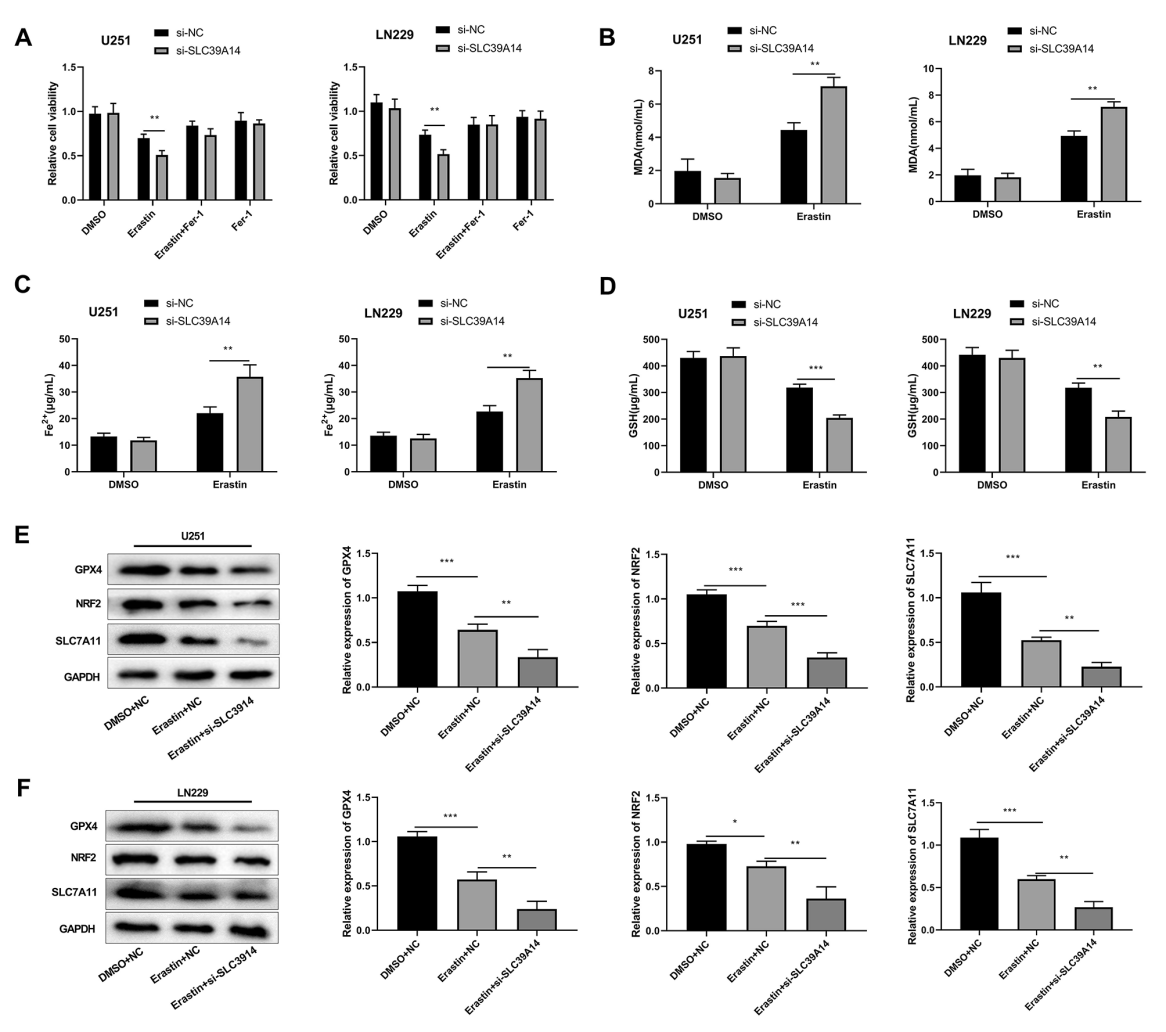
The effects of the knockdown SLC39A14 on erastin-induced ferroptosis were examined. **A**. Knockdown of SLC39A14 significantly reduced U251 and LN229 cell viability after erastin-induced ferroptosis as assessed by CCK-8. **B-D**. U251 and LN229 cells treated erastin, knockdown SLC39A14 reduced the level of MDA and Fe^2+^, while increased the level of GSH. **P < 0.01 vs. DMSO group. **E-F**. Erastin treatment caused GPX4, NRF2, and SLC7A11 protein levels to decrease in U251 and LN229 cells, and knockdown of SLC39A14 further inhibited GPX4, NRF2, and SLC7A11 protein levels. *P < 0.05; **P < 0.01; and ***P < 0.001 vs. DMSO + NC group or Erastin + NC group

Next, U251 and LN229 cells were treated with ferroptosis-activator erastin and inhibitor fer-1 to explored the effect of SLC39A14 on ferroptosis. After treatment with activator erastin, the cell proliferation capacity in the si-SLC39A14 group was remarkably lower than that in the si-NC group. However, after treatment with erastin + fer-1 or fer-1, there was no significant difference in cell viability between the si-NC group and the si-SLC39A14 group in both U251 and LN229 cells, suggesting that the knockdown of SLC39A14 had a significant impact on erastin-induced ferroptosis in glioma cells (Fig. 6A). Moreover, in both erastin-treated glioma cells, SLC39A14 knockdown significantly enhanced the concentration of MDA, Fe^2+^ and significantly reduced the levels of GSH (Fig. 6B-D).Ferroptosis is characterized by a distinctive mechanism involving GSH depletion and the accumulation of lipid ROS [7]. Downregulation of SLC7A11 and GPX4, two key regulators of ferritin deposition, decreases cystine concentration and lipid peroxide degradation, leading to the accumulation of lipid peroxides and ferroptosis [26]. Additionally, NRF2 overexpression protects malignant cells from ferroptosis [27]. Subsequently, we evaluated the impact of silencing SLC39A14 on the protein levels of SLC7A11, GPX4, and NRF2 through Western blot analysis.U251 and LN229 cells treated with erastin caused a decrease in GPX4, NRF2, SLC7A11 protein levels. And the knockdown of SLC39A14 further suppressed the protein expression levels of GPX4, NRF2, and SLC7A11 (Fig. 6E-F).

### Knockdown of SLC39A14 inhibited mouse transplantation tumor growth by promoting erastin-induced ferroptosis

**Fig. 7:**
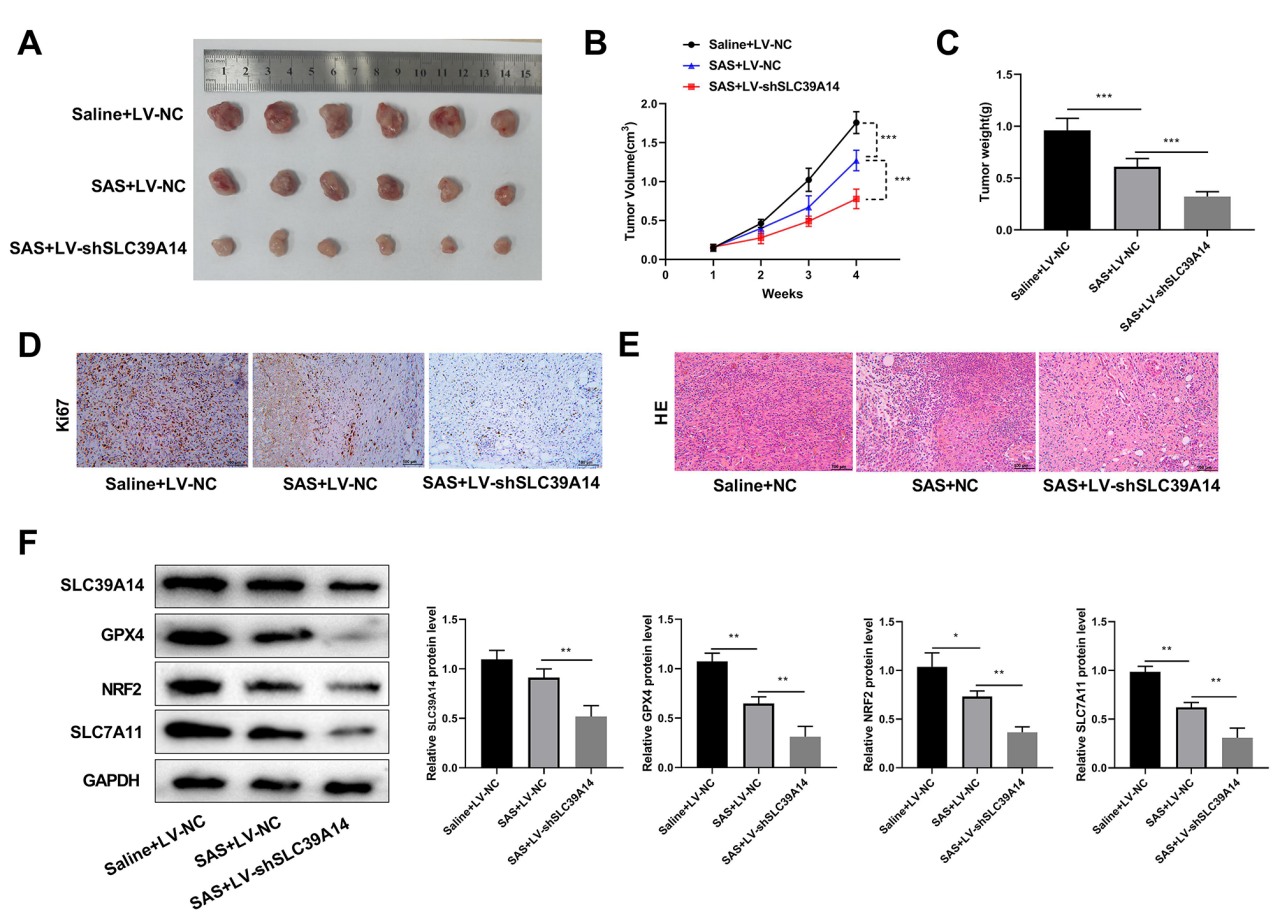
Knockdown of SLC39A14 inhibited mouse transplantation tumor growth by promoting erastin-induced ferroptosis. **(A)** Representative pictures of mouse tumors in groups Saline + LV-NC, SAS + LV-NC, and SAS + LV-shSLC39A14 (*n* = 6). **(B)** Line graphs of volume changes in mice of three groups. **(C)** Histograms displaying the weights of mice in the three groups. **D-E**. Representative IHC and HE staining of tumors from Saline + LV-NC, SAS + LV-NC, and SAS + LV-shSLC39A14 groups (scale bar for IHC = 100 μm; scale bar for HE = 50 μm). **F**. The expression levels of SLC39A14 and ferroptosis-related protein, including GPX4, NRF2 and SLC7A11 were significantly reduced when were treated with SAS and SLC39A14 knockdown. ***P < 0.001 vs. Saline + LV-NC or SAS + LV-NC.

To explore the function of SLC39A14 in vivo, a mouse transplantation tumor model was constructed. SAS was used as an in vivo ferroptosis inducer due to the nephrotoxicity of erastin. The findings indicated that the volume and weight of the mice tumors were noticeably lower in the SAS + LV-NC group compared to the Saline + LV-NC group. In addition, the mice tumor volume and weight were further decreased in the SAS + LV-shSLC39A14 group compared to the SAS + LV-NC group (Fig. 7A-C). IHC showed that Ki67 levels were reduced in the SAS + LV-NC group compared to the Saline + LV-NC group, and this trend was further enhanced by knockdown of SLC39A14 (Fig. 7D). HE results showed that SAS combined with silencing of SLC39A14 significantly reduced tumor malignancy (Fig. 7E). Western blot demonstrated SLC39A14 knockout further reduced GPX4, NRF2, and SLC7A11 protein expression by SAS-induced (Fig. 7F).

### Knockdown of SLC39A14 inhibited the cGMP-PKG signaling pathway

**Fig. 8:**
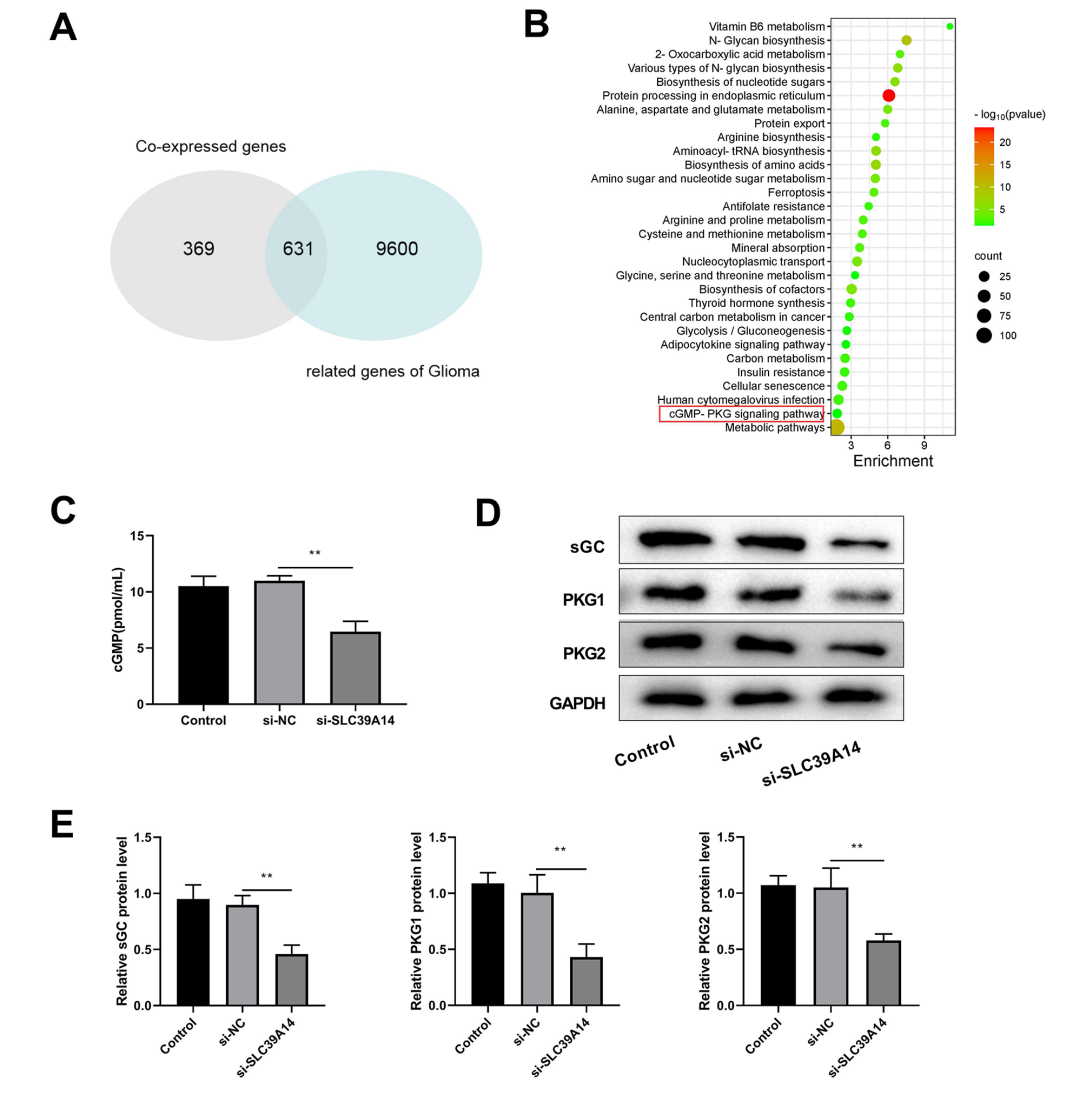
Knockdown of SLC39A14 inhibited the cGMP-PKG signaling pathway. **(A)** Venn diagram of SLC39A14 co-expressed genes and glioma-associated genes. **(B)** KEGG functional enrichment analysis of overlapping genes. On the x-axis, the number of enriched genes in this term is depicted. The term color signifies the extent of enrichment, with darker colors indicating more pronounced enrichment. **(C)** ELISA was used to detect cGMP level in Control, si-NC and si-SLC39A14 groups. **D-E**. Western blot showed that knockdown of SLC39A14 significantly inhibited the expression of the cGMP-PKG signaling pathway-related proteins, including sGC, PKG1, and PKG2. **P < 0.01 vs. Control

To investigate the molecular mechanism of SLC39A14 affecting glioma progression, the top 1000 SLC39A14 co-expressed genes and glioma-related genes were obtained from the Coxpresdb database. Then 631 shared genes were obtained by plotting Venn diagram (Fig. 8A). KEGG analysis revealed that overlapping genes were primarily highly enriched in the vitamin b6 metabolism, protein processing in endoplasmic reticulum, and n-glycan biosynthesis. Furthermore, we found that SLC39A14 might have an important correlation with the cGMP-PKG signaling pathways (Fig. 8B). Previous studies have shown that the cGMP-PKG signaling pathway plays an important role in glioma [28], but the correlation between SLC39A14 and the cGMP-PKG signaling pathways in glioma has received little attention. So, we further explored the effect of SLC39A14 on the cGMP-PKG signaling pathway. ELISA results indicated that the level of cGMP was substantially lower in the si- SLC39A14 group compared to the si-NC group (Fig. 8C). Western blot suggested that suppression of SLC39A14 significantly suppressed the cGMP-PKG pathway-associated proteins expression, including sGC, PKG1, and PKG2 (Fig. 8D-E), indicating that silencing of SLC39A14 regulate the cGMP-PKG pathway.

## Discussion

Ferroptosis, an iron-dependent regulated cell death pathway, differs significantly in morphology, genetics, and biochemistry from apoptosis, necroptosis, and autophagy [29, 30]. It has reported that ferroptosis is the predominant programmed cell death process in glioma, contributing to immunosuppression and conferring resistance to immunotherapy [31]. Nevertheless, the molecular mechanisms underlying the impact of ferroptosis on glioma progression remain elusive.

In our study, we identified seven ferroptosis-related genes through bioinformatics analysis, including SLC39A14, WWTR1, STEAP3, NOTCH2, IREB2, HIF1A, and FANCD2.WWTR1 (also known as TAZ) is a transcriptional coactivator involves in the Hippo signaling pathway and affects the progression of multiple tumors by regulating ferroptosis, including renal cell carcinoma [32, 33] and epithelial ovarian cancer [34]. In addition, several studies have shown that WWTR1 is overexpressed in gliomas [35] and promotes tumor progression through multiple pathways [36–38]. Elevated expression of STEAP3, an iron reductase in the STEAP family, enhances glioma cell migration and invasion [39, 40]. NOTCH2, a member of the Notch family, mainly contributes to promoting tumor development in gliomas [41, 42]. In addition, NOTCH2 is regulated by silencing long-chain noncoding RNA MEG8 to induce ferroptosis in hemangiomas [43]. IREB2 is involved in managing iron metabolism in cells [44]. In clear cell renal carcinoma, colorectal carcinoma and intrahepatic cholangiocarcinoma, IREB2 affects tumor development through ferroptosis-related mechanism [45–47]. HIFA, a major regulator of the hypoxic response, is activated via the prolyl hydroxylase inhibitor roxadustat, inhibiting the growth of chemotherapy resistant glioblastoma by inducing ferroptosis [48]. In low-grade gliomas, FANCD2, the ferroptosis-related gene, holds value as prognostic indicator, which can be used as a diagnostic and prognostic biomarker [49]. In this study, seven key genes were overexpressed in glioma cells and they might promote glioma progression by regulating ferroptosis. Survival analysis revealed a significant correlation between the expression of SLC39A14 and the survival of glioma patients. As a result, we selected SLC39A14 for further investigation in the follow-up study.

SLC39A14 is involved in the progression of multiple tumors. The study has shown that the overexpression of SLC39A14 in skeletal muscle contributes to the development of cachexia in metastatic malignancies [50]. Low expression of SLC39A14 in prostate cancer promotes tumor cell proliferation, invasion and migration [51]. SLC39A14 through Circ_000829 and SRSF1-mediated alternative splicing suppression, plays an anticancer role in renal cell carcinoma [52]. In the present study, we found that knockdown of SLC39A14 inhibited the proliferation, invasion and migration of glioma cells and promoted apoptosis, which has rarely been reported before. In addition, it is reported that SLC39A14 participates in the progression of multiple diseases by managing iron metabolism. Knockout of SLC39A14 expression in Trf-LKO mice effectively decreases hepatic iron accumulation, consequently reducing ferritin deposition-associated hepatic fibrosis induced by a high-iron diet or CCl4 injection [53]. Furthermore, elevated levels of SLC39A14 protein at the cell membrane significantly correlated with the accumulation of labile iron in skeletal muscle of aged rodents [54]. In tumors, SLC39A14 can be used as a ferroptosis-related biomarker [22]. Silencing SLC39A14 enhanced erastin-induced ferroptosis, as evidenced by elevated MDA and Fe^2+^ levels, along with a marked reduction in GSH. Additionally, erastin treatment increased the protein levels of GPX4, NRF2, and SLC7A11, which could be further attenuated by silencing SLC39A14.

Erastin, the prototype ferroptosis inducer, can inhibit the xc-system to decrease GSH levels, promote molecular chaperone-mediated autophagy, degrade GPX4, and induce mitochondrial dysfunction [55, 56]. In the current investigation, we found that SLC39A14 knockdown promoted erastin-induced ferroptosis. Ferroptosis primarily results from the disruption of cellular antioxidant systems, notably the system xc − GSH-GPX4-dependent defense mechanism, leading to the buildup of lipid hydroperoxides [57]. MDA is a biomarker for oxidative stress and lipid peroxidation [58]. SLC7A11 is a functional component of the system xc-, which is overexpressed in various human cancers. It can suppress ferroptosis via importing cystine, boosting GSH production, and enabling GPX4-mediated lipid peroxide detoxification, which in turn promotes tumor growth [59, 60]. In glioma, hypoxia augments tumor resistance to Sulfasalazine-induced ferroptosis by increasing SLC7A11 expression through activation of the PI3K/AKT/HIF-1α pathway [61]. NRF2, a crucial transcription factor, plays a vital role in inhibiting ferroptosis by suppressing cellular iron uptake, reducing ROS generation, and upregulating the expression of SLC7A11 [62]. In glioma, the NRF2-Keap1 pathway stimulates cell proliferation while reducing ferroptosis [63]. GPX4 is a well-known ferroptosis gatekeeper that plays an important function in regulating lipid peroxidation [64]. Moreover, GPX4 counteracts ferroptosis by utilizing reduced GSH as a cofactor to detoxify lipid peroxides into lipols [65]. In glioblastoma, inhibition of GPX4 expression by RSL3 induces ferroptosis [66]. Previous study has shown that SLC39A14 functions as a transmembrane transporter responsible for transporting non-transferrin-bound iron, specifically ferrous iron [67]. In the present investigation, we found that SLC39A14 silencing may promote erastin-induced ferroptosis in glioma by modulating the levels of MDA, Fe^2+^, GSH, GPX4, NRF2, and SLC7A11, which may further inhibit the progression of gliomas.

Subsequently, we investigated the mechanisms through which SLC39A14 influences glioma progression. The results showed that SLC39A14 had an important correlation with the cGMP-PKG pathway.Multiple studies have shown that the cGMP-PKG pathway promotes tumor progression. In cervical cancer, the cGMP-PKG pathway is activated by the upregulation of lncRNA DARS-AS1, which accelerates tumor malignancy [68]. Nucleotide de novo synthesis enhances breast cancer stemness and metastasis through the cGMP-PKG-MAPK signaling pathway activation [69]. In gliomas, the enhancement of stem cell-like characteristics in the tumor PVN by the NO/cGMP/PKG pathway could potentially reveal therapeutic targets [28]. In our study, we found that silencing SLC39A14 decreased the concentration of cGMP and the protein expression levels of sGC, PKG1 and PKG2. The correlation between SLC39A14 and the cGMP-PKG pathway has rarely been reported in tumors. In other diseases, SLC39A14 upregulation activates NO, a key regulator of the cGMP-PKG pathway, mediating hepatic zinc accumulation and hypozincemia during inflammation and sepsis [70, 71]. Phosphodiesterase, responsible for degradation of cGMP, is inhibited by SLC39A14, mediating systemic growth [72, 73]. Our research shows that SLC39A14 may play a role in glioma progression by modulating the cGMP-PKG signaling pathway.

There were also some limitations in this study. Firstly, the GSE15209 and GSE31262 datasets, which were utilized for the bioinformatics analyses in this study, did not contain very rich tumor and normal sample sizes. Then, using bioinformatics survival analysis exclusively, we screened SLC39A14 from the seven key genes for further investigation and did not analyze the remaining genes. Furthermore, after knocking down SLC39A14, we only measured the expression of a few key factor in the cGMP-PKG signaling pathway. Further experimental exploration is required to establish the correlation between SLC39A14 and the cGMP-PKG signaling pathway and their roles in glioma. In conclusion, we identify seven ferroptosis-associated genes (SLC39A14, WWTR1, STEAP3, NOTCH2, IREB2, HIF1A, and FANCD2) in gliomas, all of which are highly expressed. Knockdown of SLC39A14 inhibits glioma cell growth, metastasis and promotes apoptosis. In addition, knockdown of SLC39A14 promotes erastin-induced ferroptosis, which in turn inhibits the growth of mouse transplantation tumors by a mechanism that may be attributed to the regulation of the cGMP-PKG signaling pathway.

### Electronic supplementary material

Below is the link to the electronic supplementary material.

Supplementary Material 1

Supplementary Material 2

## Data Availability

The GSE15209 and GSE31262 datasets analysed during the current study are available in the GEO DataSets repository, [https://www.ncbi.nlm.nih.gov/geo/], under accession numbers GSE15209 and GSE31262.
