# Supplementary material for: Knockdown of SLC39A14 inhibits glioma progression by promoting erastin-induced ferroptosis SLC39A14 knockdown inhibits glioma progression
Source: BMC Cancer. 2023 Nov 17;23:1120. doi: 10.1186/s12885-023-11637-0 (PMC10655456; doi:10.1186/s12885-023-11637-0)
Supplement: Supplementary file 1 — Supplementary Material 1 [file 12885_2023_11637_MOESM1_ESM.docx]

**
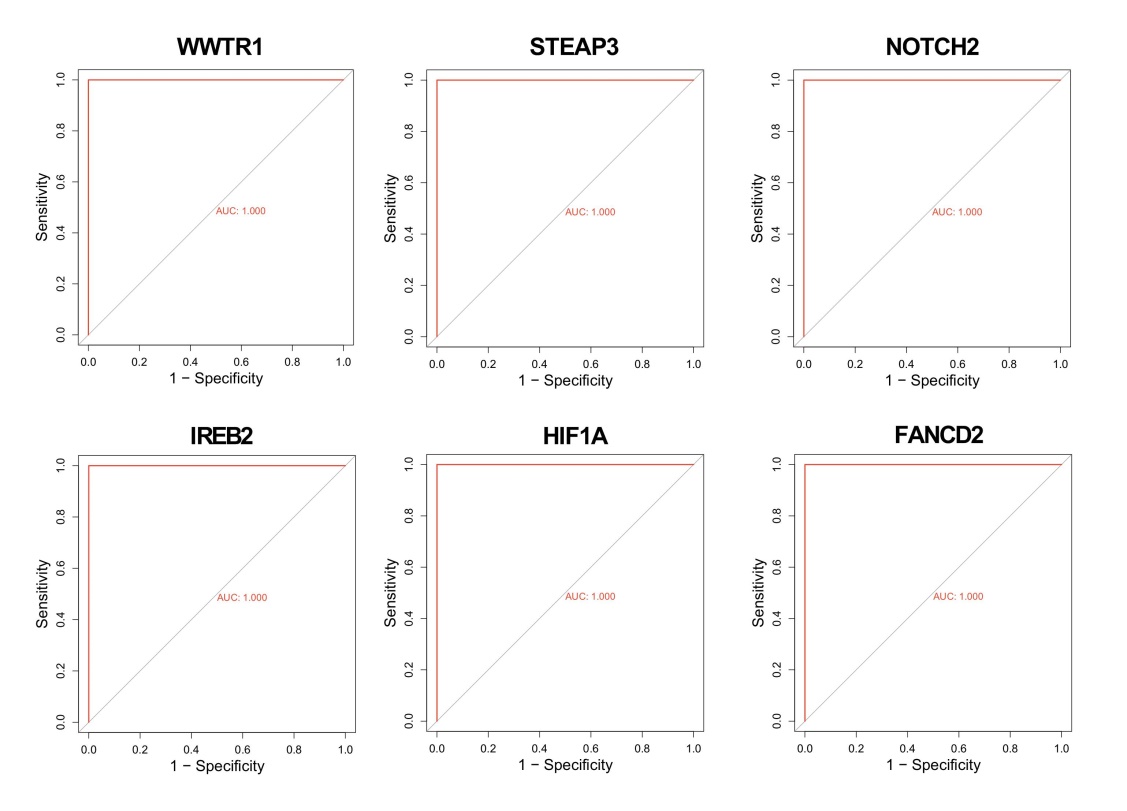
**

**Supplementary figure 1** Supplementary Figure 1 ROC curves for WWTR1, STEAP3, NOTCH2, IREB2, HIF1A, and FANCD2.


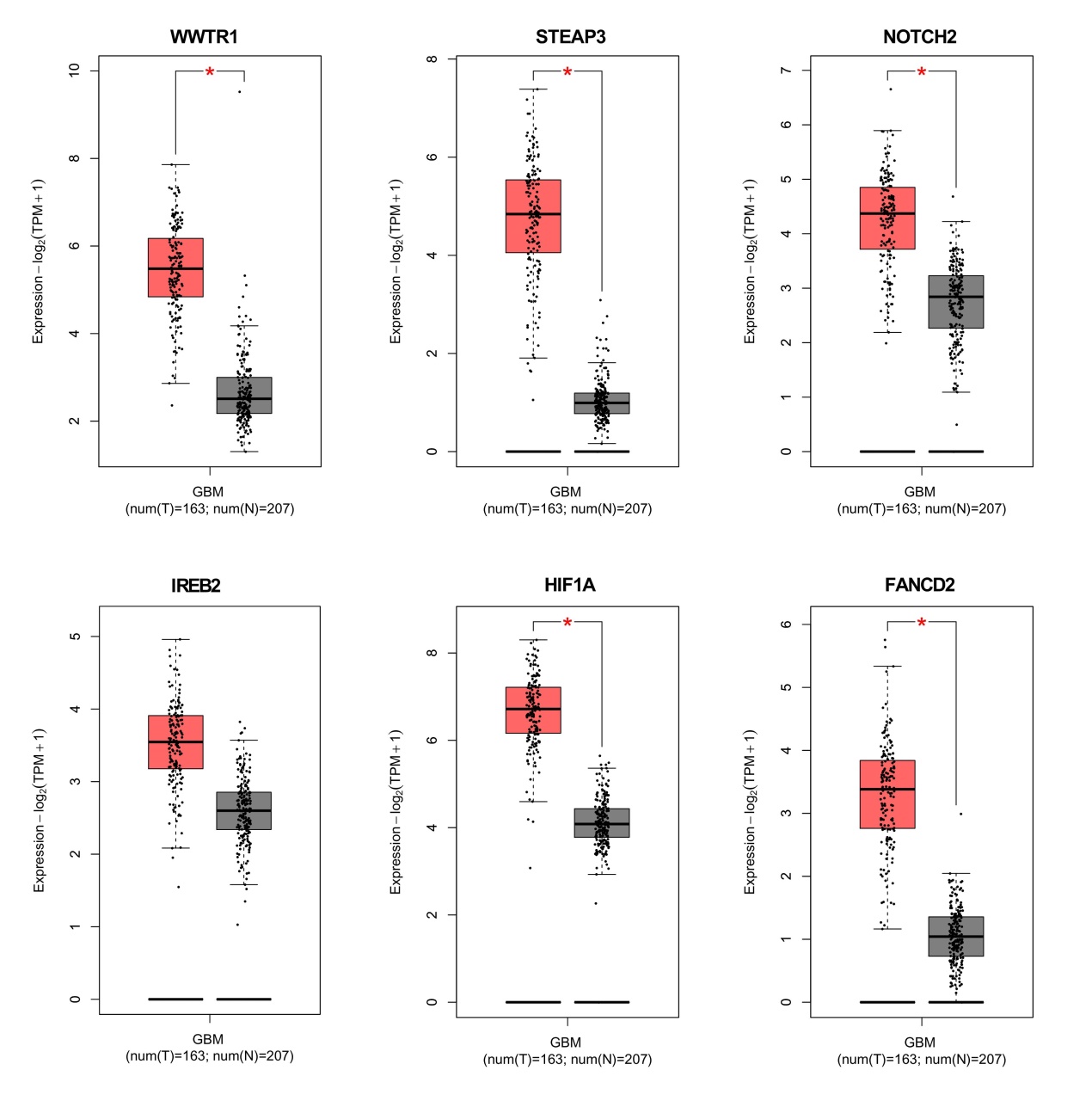


**Supplementary figure 2** The mRNA expression levels of WWTR1, STEAP3, NOTCH2, IREB2, HIF1A, and FANCD2 in glioma tissues analyzed in the GEPIA 2 database.


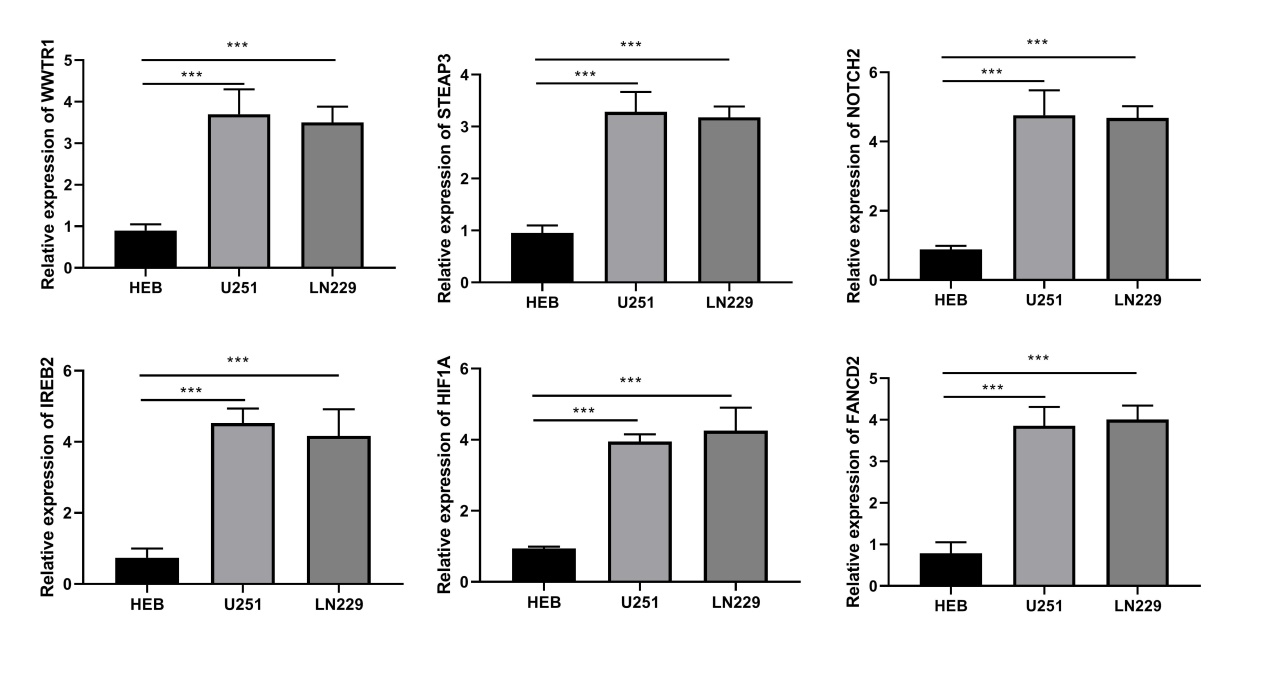


**Supplementary figure 3** RT-qPCR was used to assess the mRNA expression levels of WWTR1, STEAP3, NOTCH2, IREB2, HIF1A, and FANCD2 in glioma cells. ***P < 0.001 vs. HEB group.

**Supplementary Table 1** Sequence information used in this study

| Name | Sequences（5’-3’） |
| --- | --- |
| si-NC-F | UUCUCCGAACGUGUCACGUTT |
| si-NC-R | ACGUGACACGUUCGGAGAATT |
| si-SLC39A14-1-F | GGAGUAGAAUACUAAGUUAGA |
| si-SLC39A14-1-R | UAACUUAGUAUUCUACUCCAA |
| si-SLC39A14-2-F | GGAGGAAUGUUCUUGUAUAUU |
| si-SLC39A14-2-R | UAUACAAGAACAUUCCUCCAG |
| si-SLC39A14-3-F | GGUGUAUGAGAAGAAAUUAGA |
| si-SLC39A14-3-R | UAAUUUCUUCUCAUACACCUG |
| GAPDH-F | GAGTCAACGGATTTGGTCGT |
| GAPDH-R | TTGATTTTGGAGGGATCTCG |
| SLC39A14-F | GCTGTCTAACGCGCTATTCC |
| SLC39A14-R | AATGGCTGTGTCCATGATGA |
| WWTR1-F | CAGCAAATGTGGATGAGATGG |
| WWTR1-R | TCATTGAAGAGGGGGATCAG |
| STEAP3-F | CTTTCCAAGAGGAGGCAGTG |
| STEAP3-R | TTGGACTCACGATGCTGAAG |
| NOTCH2-F | AAGCAGAGTCCCAGTGCCTA |
| NOTCH2-R | CAGGGGGCACTGACAGTAAT |
| IREB2-F | ATTCGGCAGAAATCGAGAGA |
| IREB2-R | CCCCAGAATCCCTAAACCAT |
| HIF1A-F | TCAAGTCAGCAACGTGGAAG |
| HIF1A-R | TATCGAGGCTGTGTCGACTG |
| FANCD2-F | TCAGACCCTGAGGAGACACC |
| FANCD2-R | ATGTCAATCCCCAGAAGCAG |

**Supplementary Table 2** Top 15 up- and down-regulated DEGs in the GSE15209 dataset

| **Name** | **Description** | **log2FoldChange** | **pval** | **up/down** |
| --- | --- | --- | --- | --- |
| CTSC | cathepsin C | 4.84 | 2.96E-10 | up |
| POSTN | periostin | 4.79 | 5.50E-07 | up |
| TOP2A | topoisomerase (DNA) II alpha | 4.67 | 1.97E-07 | up |
| CALD1 | caldesmon 1 | 4.23 | 6.81E-10 | up |
| ITGB1 | integrin subunit beta 1 | 4.22 | 3.01E-10 | up |
| PTX3 | pentraxin 3 | 4.22 | 1.40E-04 | up |
| POSTN | periostin | 4.21 | 3.99E-06 | up |
| CALU | calumenin | 4.15 | 1.50E-13 | up |
| TOP2A | topoisomerase (DNA) II alpha | 4.04 | 1.72E-07 | up |
| CDK4 | cyclin dependent kinase 4 | 4.04 | 3.28E-06 | up |
| ITGB1 | integrin subunit beta 1 | 4.02 | 9.30E-10 | up |
| P4HB | prolyl 4-hydroxylase subunit beta | 4.01 | 3.56E-11 | up |
| IGF2BP3 | insulin like growth factor 2 mRNA binding protein 3 | 4.01 | 2.57E-07 | up |
| TFPI2 | tissue factor pathway inhibitor 2 | 3.95 | 1.30E-04 | up |
| IGF2BP3 | insulin like growth factor 2 mRNA binding protein 3 | 3.94 | 3.20E-07 | up |
| HBA2///HBA1 | hemoglobin subunit alpha 2///hemoglobin subunit alpha 1 | -6.19 | 2.07E-09 | down |
| SLC1A2 | solute carrier family 1 member 2 | -6.1 | 6.64E-10 | down |
| AQP4 | aquaporin 4 | -6.05 | 6.21E-09 | down |
| RTN1 | reticulon 1 | -5.96 | 1.47E-08 | down |
| HBB | hemoglobin subunit beta | -5.95 | 3.78E-08 | down |
| HBB | hemoglobin subunit beta | -5.91 | 1.11E-08 | down |
| FAM107A | family with sequence similarity 107 member A | -5.61 | 9.00E-13 | down |
| PRKCB | protein kinase C beta | -5.39 | 1.01E-11 | down |
| HBA2///HBA1 | hemoglobin subunit alpha 2///hemoglobin subunit alpha 1 | -5.39 | 6.66E-08 | down |
| ATP1A2 | ATPase Na+/K+ transporting subunit alpha 2 | -5.38 | 2.48E-11 | down |
| VSNL1 | visinin like 1 | -5.31 | 1.12E-11 | down |
| HBA2///HBA1 | hemoglobin subunit alpha 2///hemoglobin subunit alpha 1 | -5.17 | 1.86E-08 | down |
| GABRB2 | gamma-aminobutyric acid type A receptor beta2 subunit | -5.13 | 6.37E-11 | down |
| PRKCB | protein kinase C beta | -5.1 | 2.63E-12 | down |
| MBP | myelin basic protein | -5.08 | 1.34E-05 | down |

**Supplementary Table 3** Top 15 up- and down-regulated DEGs in the GSE31262 dataset

| **Name** | **Description** | **log2FoldChange** | **pval** | **up/down** |
| --- | --- | --- | --- | --- |
| IGF2BP3 | insulin like growth factor 2 mRNA binding protein 3 | 6.54 | 4.67E-06 | up |
| TOP2A | topoisomerase (DNA) II alpha | 5.43 | 4.33E-08 | up |
| HOXA10 | homeobox A10 | 5.18 | 5.10E-05 | up |
| CENPA | centromere protein A | 5.02 | 6.81E-08 | up |
| DLGAP5 | DLG associated protein 5 | 4.90 | 2.11E-08 | up |
| CCDC80 | coiled-coil domain containing 80 | 4.90 | 2.48E-05 | up |
| NCAPG | non-SMC condensin I complex subunit G | 4.88 | 8.33E-05 | up |
| EGFR | epidermal growth factor receptor | 4.86 | 9.34E-04 | up |
| TEAD2 | TEA domain transcription factor 2 | 4.85 | 7.33E-05 | up |
| DLX1 | distal-less homeobox 1 | 4.80 | 4.00E-04 | up |
| PBK | PDZ binding kinase | 4.80 | 2.93E-06 | up |
| FILIP1L | filamin A interacting protein 1 like | 4.79 | 5.58E-06 | up |
| BIRC5 | baculoviral IAP repeat containing 5 | 4.70 | 1.03E-04 | up |
| EN1 | engrailed homeobox 1 | 4.62 | 1.25E-03 | up |
| CENPF | centromere protein F | 4.59 | 5.30E-05 | up |
| ITM2A | integral membrane protein 2A | -8.28 | 4.30E-07 | down |
| MAG | myelin associated glycoprotein | -7.76 | 1.23E-06 | down |
| CNDP1 | carnosine dipeptidase 1 | -7.74 | 2.78E-05 | down |
| CAPN3 | calpain 3 | -7.53 | 1.08E-07 | down |
| RBP7 | retinol binding protein 7 | -7.46 | 1.54E-07 | down |
| NKX6-2 | NK6 homeobox 2 | -7.44 | 2.93E-07 | down |
| BCAS1 | breast carcinoma amplified sequence 1 | -7.44 | 2.57E-05 | down |
| TF | transferrin | -7.39 | 3.28E-06 | down |
| ENPP2 | ectonucleotide pyrophosphatase/phosphodiesterase 2 | -7.28 | 1.89E-05 | down |
| FOLH1B | folate hydrolase 1B | -7.23 | 2.55E-08 | down |
| PPP1R14A | protein phosphatase 1 regulatory inhibitor subunit 14A | -7.08 | 1.11E-07 | down |
| CNTNAP4 | contactin associated protein like 4 | -7.07 | 8.37E-08 | down |
| MOG | myelin oligodendrocyte glycoprotein | -7.03 | 2.29E-06 | down |
| MBP | myelin basic protein | -6.86 | 3.33E-06 | down |
| PTGDS | prostaglandin D2 synthase | -6.84 | 3.88E-07 | down |

**Supplementary Table 4** GO for DEGs

| Category | Term | Genes | Count | PValue | Fold Enrichment |
| --- | --- | --- | --- | --- | --- |
| BP | heart process | YAP1, WWTR1 | 2 | 1.51E-02 | 128.72 |
| BP | regulation of metanephric nephron tubule epithelial cell differentiation | YAP1, WWTR1 | 2 | 1.51E-02 | 128.72 |
| BP | negative regulation of cardiac muscle cell differentiation | FZD7, DKK1 | 2 | 2.26E-02 | 85.81 |
| BP | negative regulation of cell adhesion mediated by integrin | SNAI2, JAM3 | 2 | 2.51E-02 | 77.23 |
| BP | Notch signaling involved in heart development | NOTCH2, SNAI2 | 2 | 2.76E-02 | 70.21 |
| BP | positive regulation of intracellular estrogen receptor signaling pathway | YAP1, SKP2 | 2 | 3.25E-02 | 59.41 |
| CC | autolysosome | LAMP2, FTL | 2 | 2.59E-02 | 74.77 |
| CC | chaperone complex | RUVBL1, DNAJC9 | 2 | 6.91E-02 | 27.42 |
| CC | mitotic spindle | MAPK1, MAP4, TUBB4A, AURKA | 4 | 4.53E-03 | 11.75 |
| CC | growth cone | COPA, RUFY3, ENO2, SNCA | 4 | 5.38E-03 | 11.04 |
| CC | microtubule organizing center | RPS7, RUVBL1, MAPK1, MAP4 | 4 | 6.56E-03 | 10.28 |
| CC | microtubule cytoskeleton | PRC1, MAP4, TUBB4A, AURKA | 4 | 1.15E-02 | 8.35 |
| MF | histone serine kinase activity | PRKAA1, AURKA | 2 | 1.27E-02 | 154.03 |
| MF | ferric iron binding | TF, FTL | 2 | 3.75E-02 | 51.34 |
| MF | ferrous iron binding | TF, FTL, SNCA | 3 | 2.30E-03 | 41.26 |
| MF | DNA polymerase binding | PCNA, FANCD2 | 2 | 5.45E-02 | 35.01 |
| MF | histone acetyltransferase binding | PCNA, SP1 | 2 | 6.17E-02 | 30.81 |
| MF | double-stranded DNA binding | SP1, MAPK1, EGFR | 3 | 3.57E-02 | 9.87 |

**Supplementary Table 5** KEGG for DEGs

| Category | Term | Genes | Count | PValue | Fold Enrichment |
| --- | --- | --- | --- | --- | --- |
| KEGG_PATHWAY | Ferroptosis | STEAP3, TF, SLC39A14, FTL | 4 | 5.78E-04 | 23.43 |
| KEGG_PATHWAY | HIF-1 signaling pathway | TF, PRKCB, MAPK1, ENO2, HIF1A, EGFR | 6 | 6.82E-05 | 13.22 |
| KEGG_PATHWAY | Choline metabolism in cancer | SP1, PRKCB, MAPK1, HIF1A, EGFR | 5 | 6.12E-04 | 12.25 |
| KEGG_PATHWAY | Gap junction | PRKCB, MAPK1, TUBB4A, EGFR | 4 | 5.23E-03 | 10.91 |
| KEGG_PATHWAY | Central carbon metabolism in cancer | MAPK1, HIF1A, EGFR | 3 | 3.23E-02 | 10.29 |
| KEGG_PATHWAY | Adherens junction | MAPK1, SNAI2, EGFR | 3 | 3.31E-02 | 10.15 |
| KEGG_PATHWAY | Non-small cell lung cancer | PRKCB, MAPK1, EGFR | 3 | 3.40E-02 | 10.00 |
| KEGG_PATHWAY | Endocrine resistance | NOTCH2, SP1, MAPK1, EGFR | 4 | 7.05E-03 | 9.80 |
| KEGG_PATHWAY | Glioma | PRKCB, MAPK1, EGFR | 3 | 3.66E-02 | 9.60 |
| KEGG_PATHWAY | EGFR tyrosine kinase inhibitor resistance | PRKCB, MAPK1, EGFR | 3 | 4.02E-02 | 9.12 |
