# Supplementary material for: Knockdown of SLC39A14 inhibits glioma progression by promoting erastin-induced ferroptosis SLC39A14 knockdown inhibits glioma progression
Source: BMC Cancer. 2023 Nov 17;23:1120. doi: 10.1186/s12885-023-11637-0 (PMC10655456; doi:10.1186/s12885-023-11637-0)

Figure6E-U251-GPX4

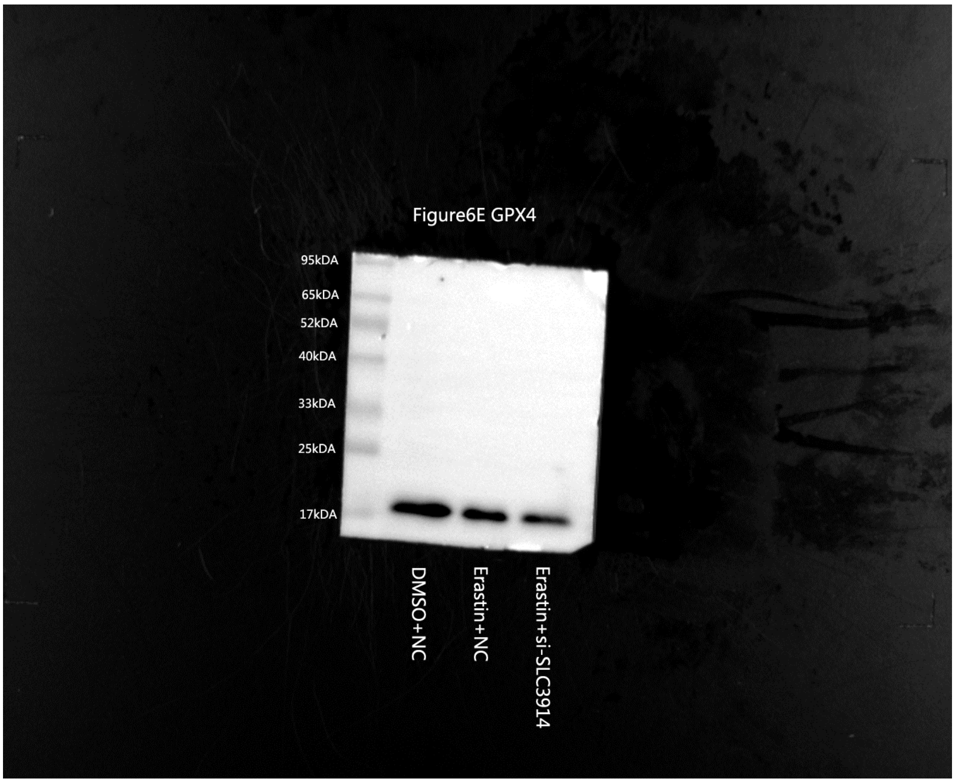

Figure6E-U251-NRF2

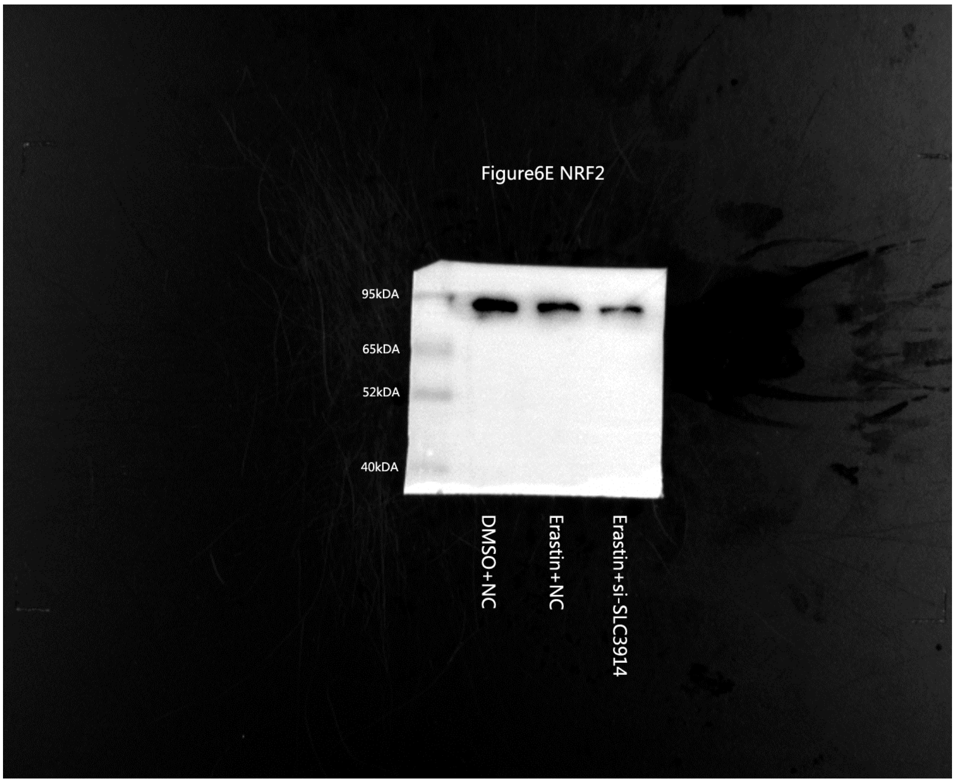

Figure6E-U251-SLC7A11

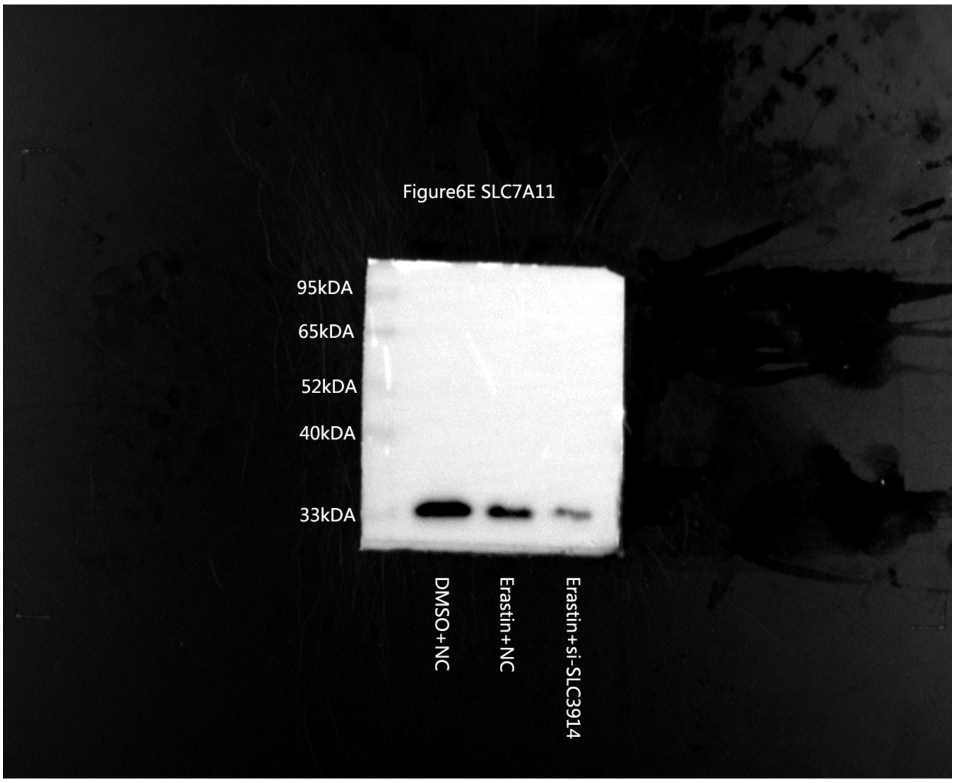

Figure6E-U251-GAPDH

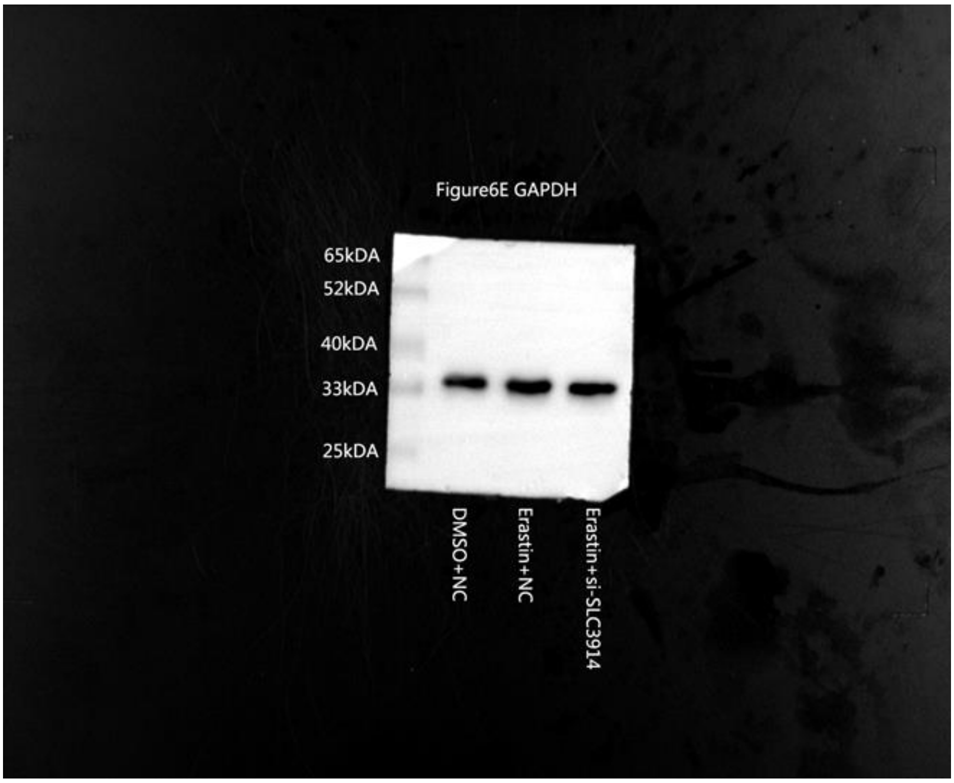

Figure6F-LN229-GPX4

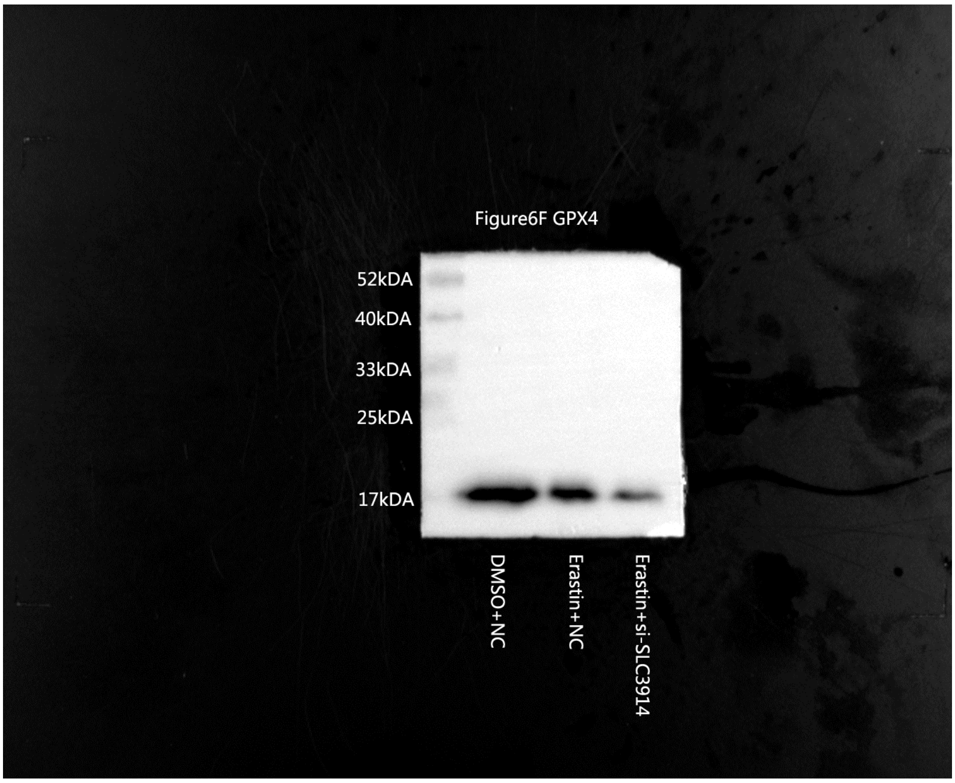

Figure6F-LN229-NRF2

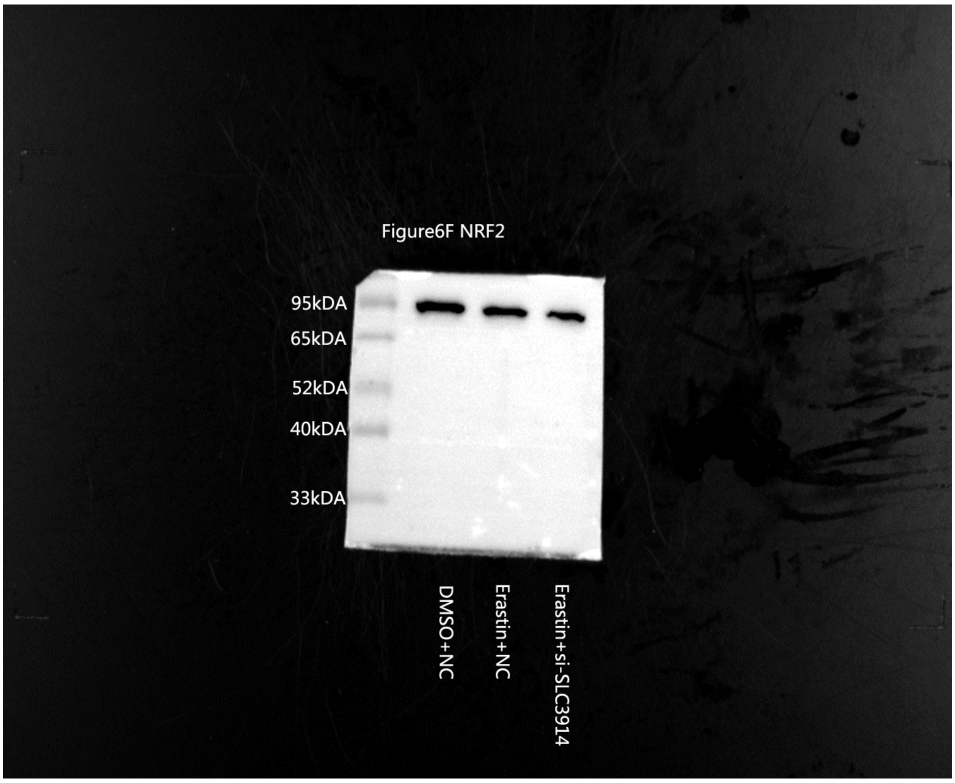

Figure6F-LN229-SLC7A11

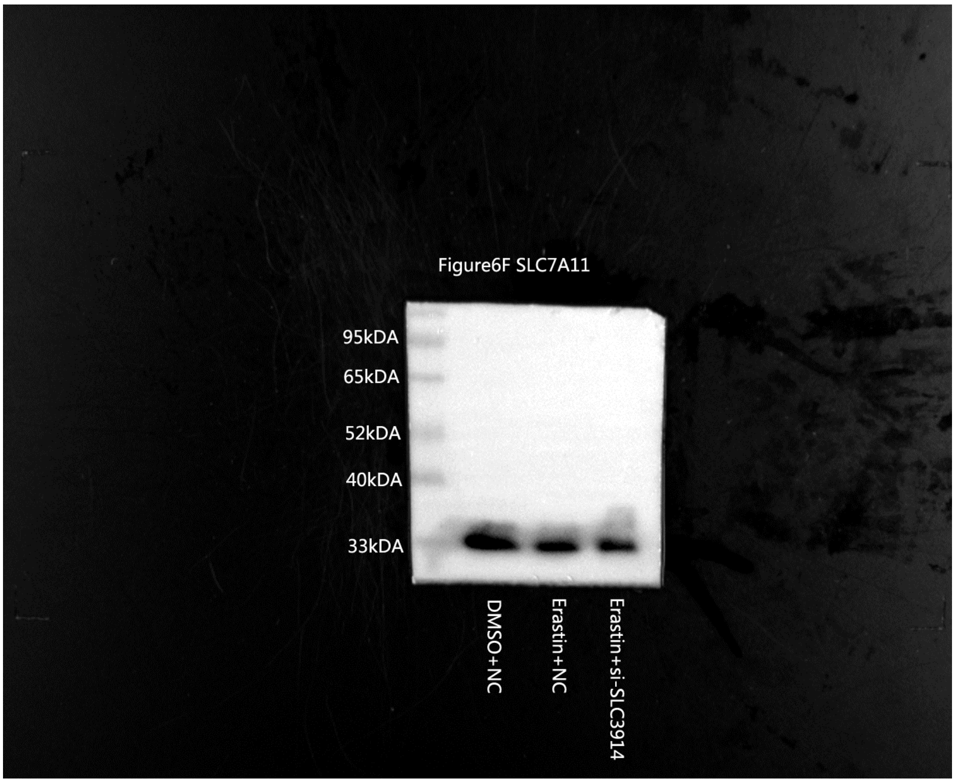

Figure6F-LN229-GAPDH

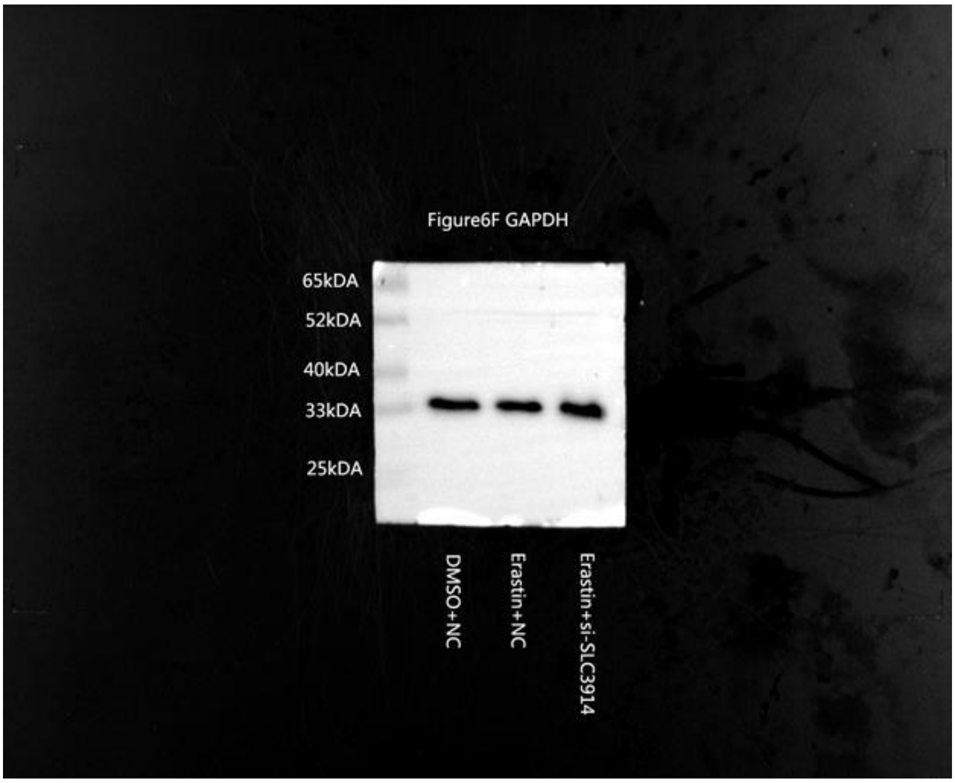

Figure7F –SLC39A14

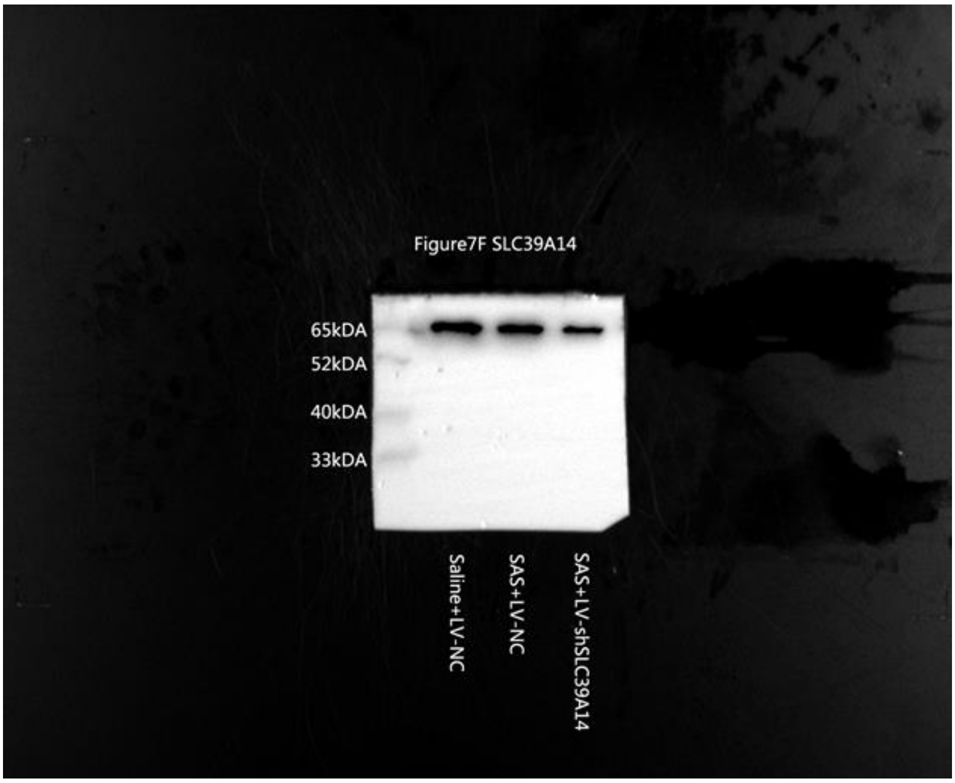

Figure7F –GPX4

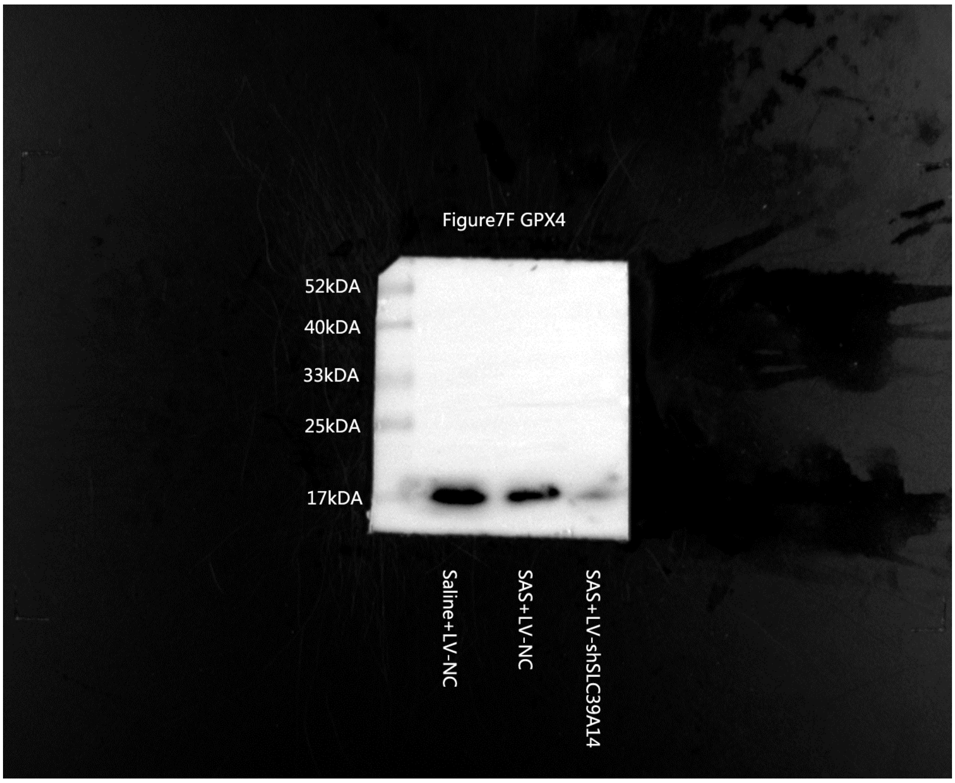

Figure7F –NRF2

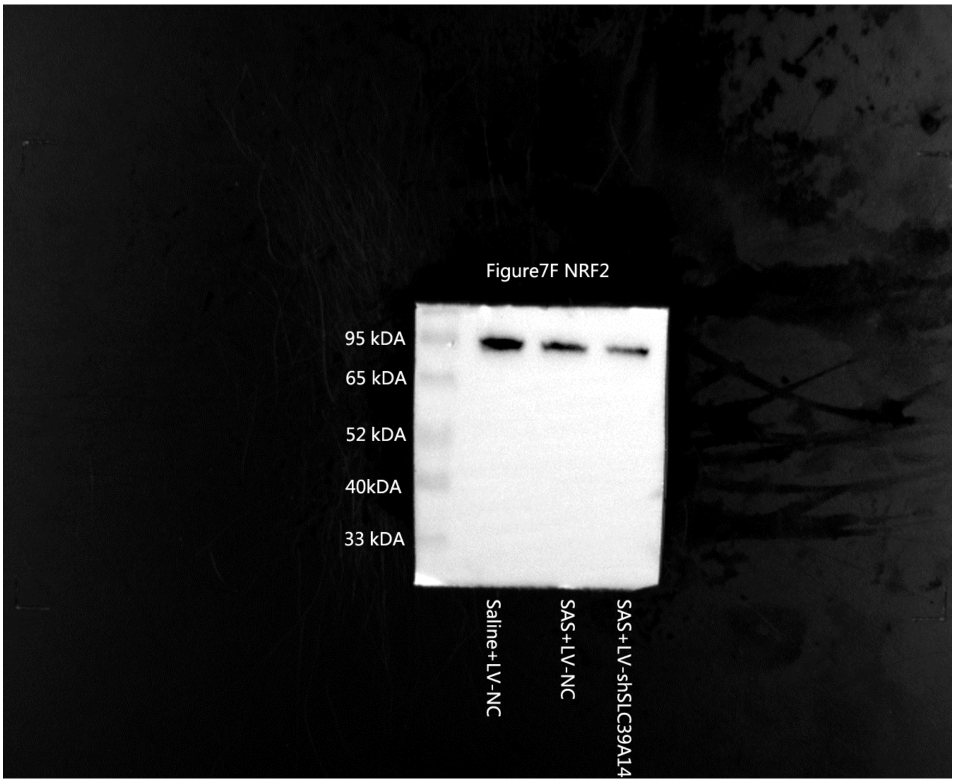

Figure7F –SLC7A11

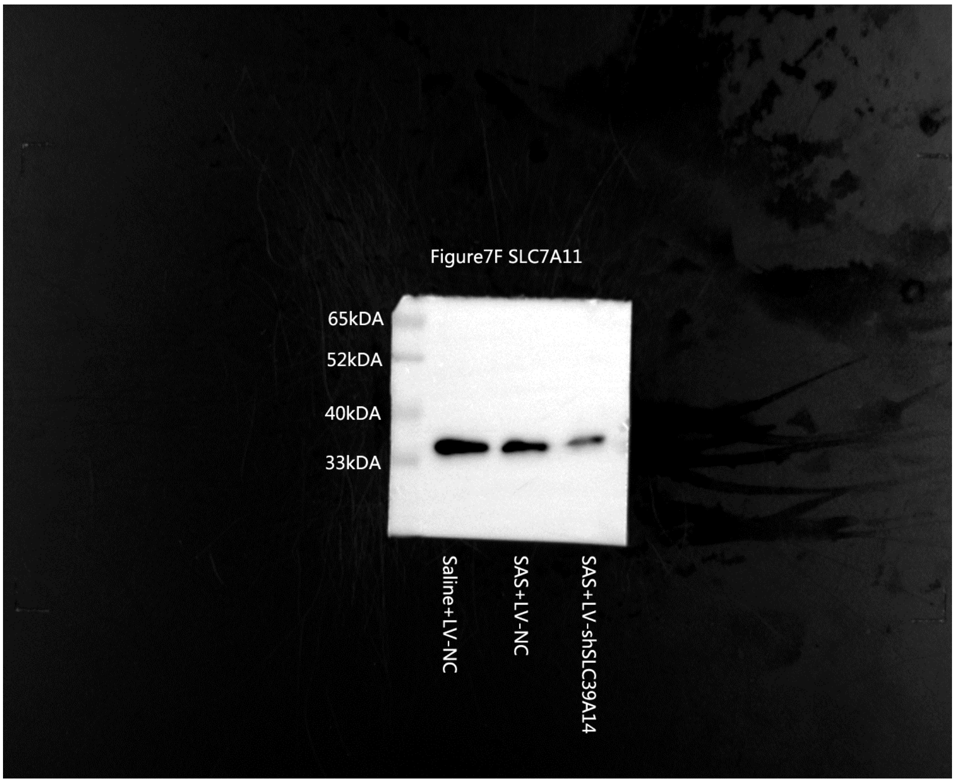

Figure7F -GAPDH

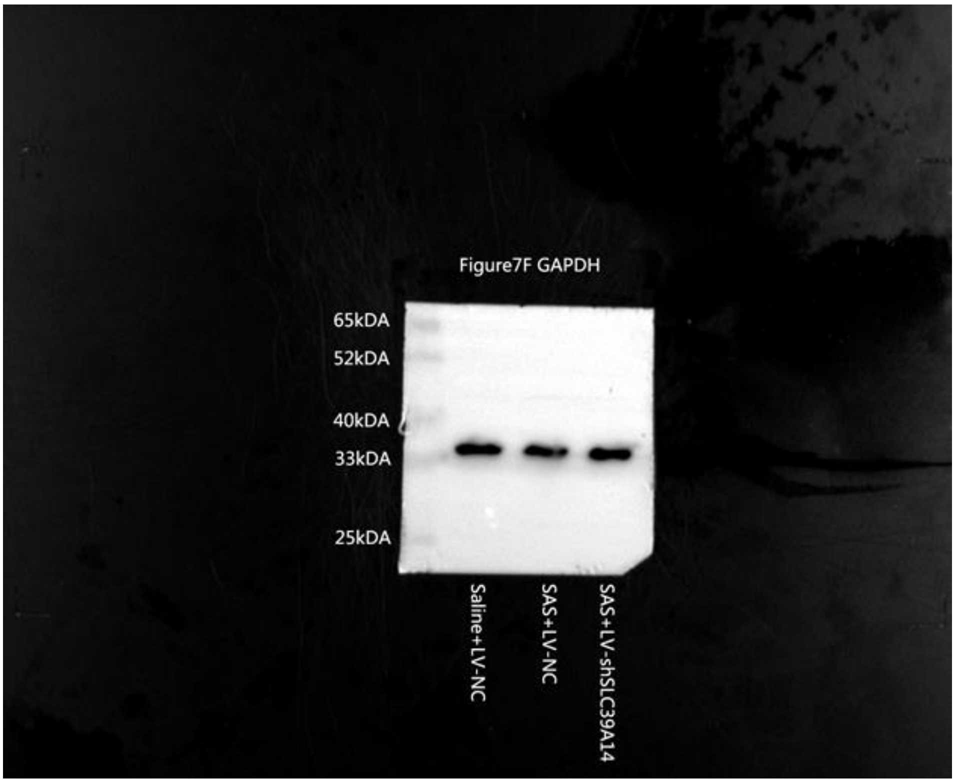

Figure8D –sGC

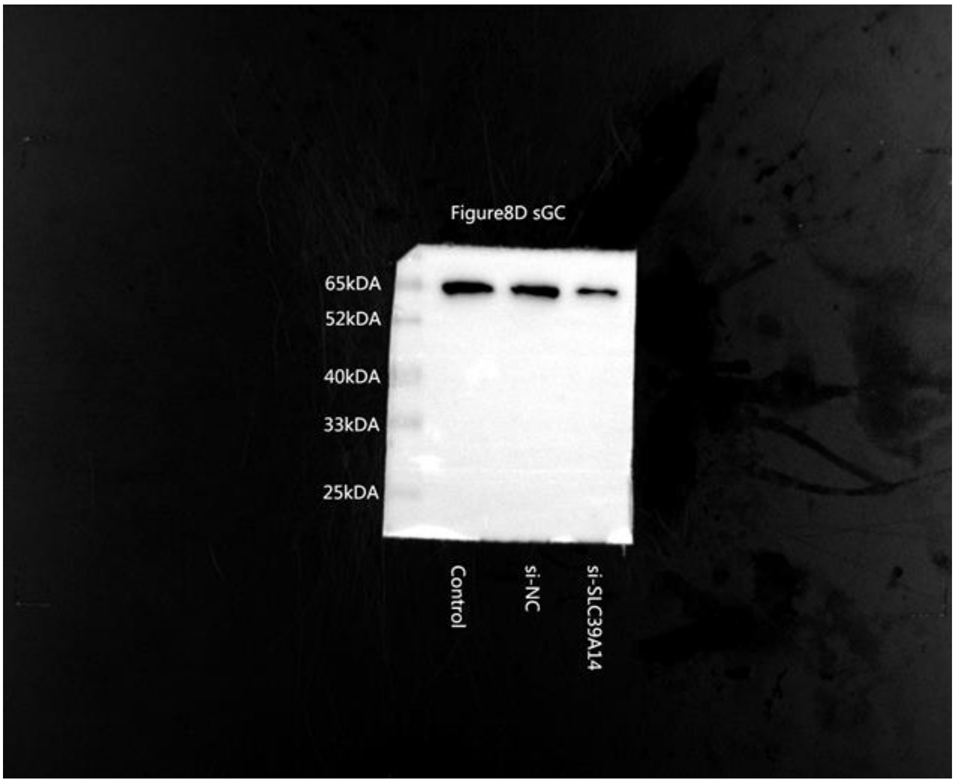

Figure8D –PKG1

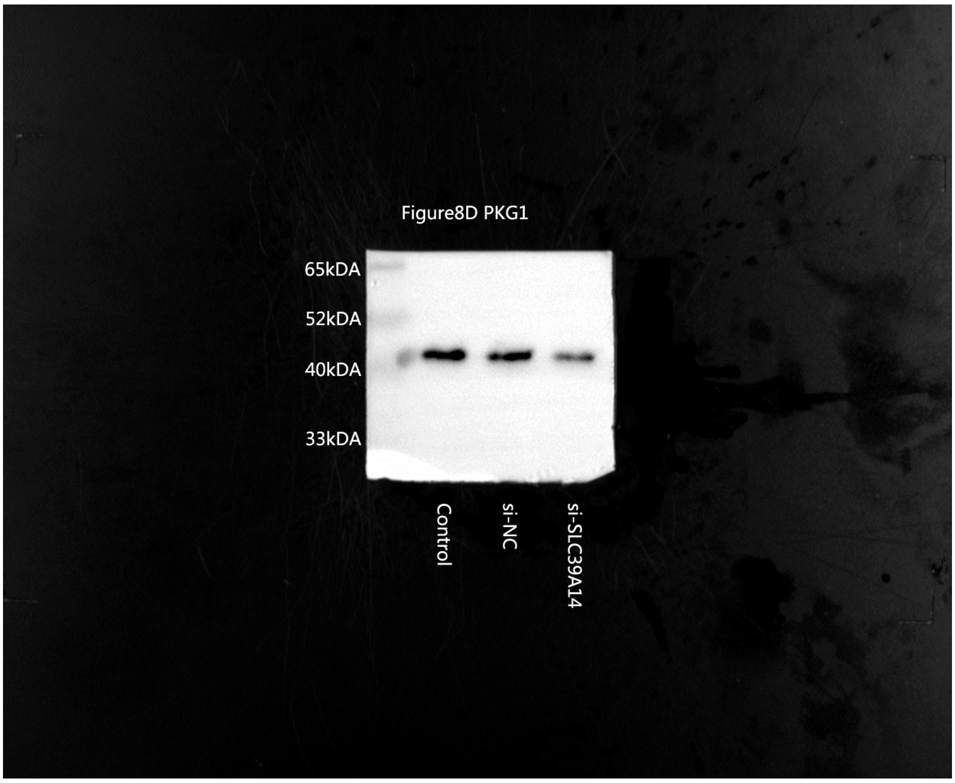

Figure8D –PKG2

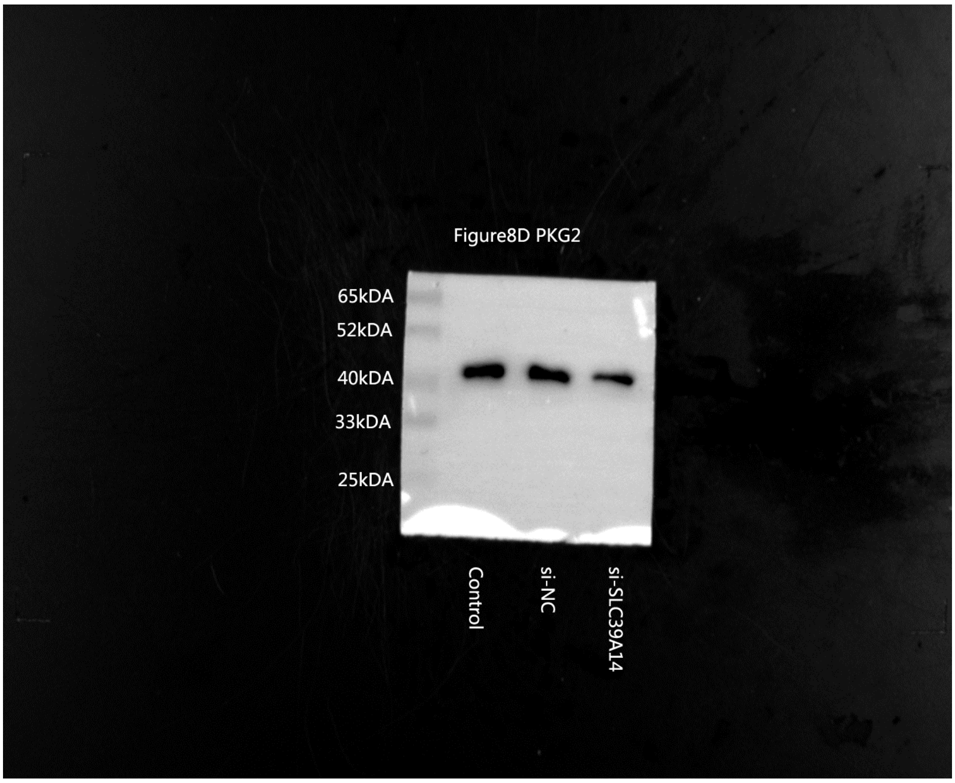

Figure8D –GAPDH

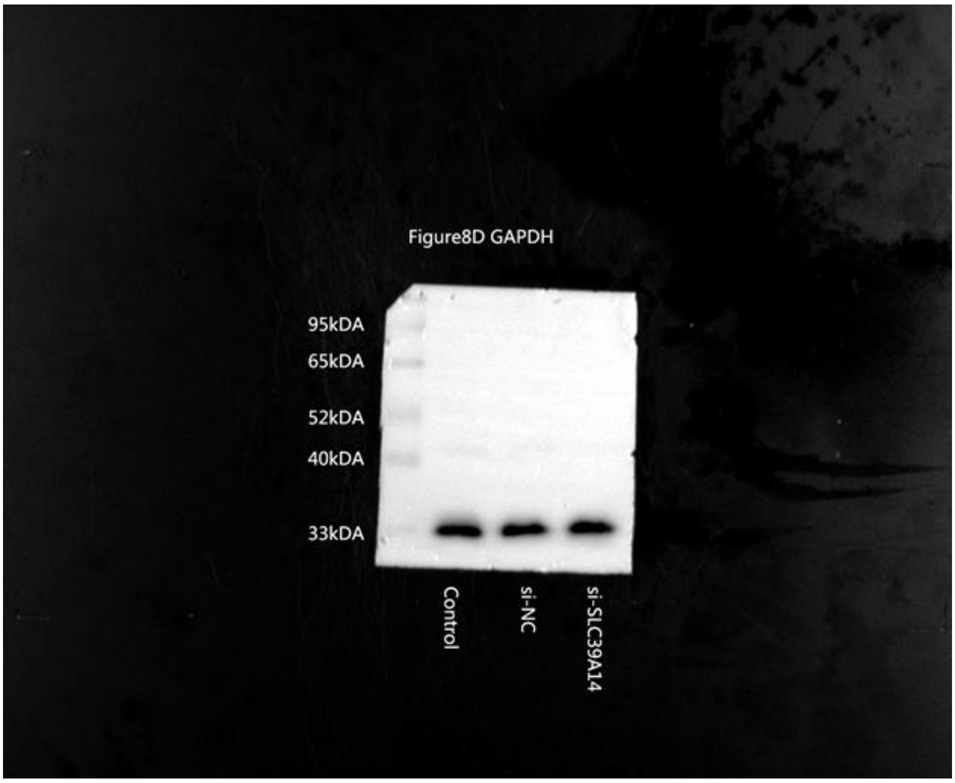

Supplement: Supplementary file 2 — Supplementary Material 2 [file 12885_2023_11637_MOESM2_ESM.pdf]
